# Landscape of brain myeloid cell transcriptome along the spatiotemporal progression of Alzheimer’s disease reveals distinct sequential responses to Aβ and tau

**DOI:** 10.1007/s00401-024-02704-2

**Published:** 2024-04-01

**Authors:** Astrid Wachter, Maya E. Woodbury, Sylvia Lombardo, Aicha Abdourahman, Carolin Wuest, Emily McGlame, Timothy Pastika, Joseph Tamm, Nandini Romanul, Kiran Yanamandra, Rachel Bennett, Gen Lin, Taekyung Kwon, Fan Liao, Corinna Klein, Yelena Grinberg, Methasit Jaisa-aad, Huan Li, Matthew. P. Frosch, Markus P. Kummer, Sudeshna Das, Tammy Dellovade, Eric H. Karran, Xavier Langlois, Janina S. Ried, Alberto Serrano-Pozo, Robert V. Talanian, Knut Biber, Bradley T. Hyman

**Affiliations:** 1 AbbVie Deutschland GmbH & Co. KG, Ludwigshafen, Germany; 2 AbbVie Inc., Cambridge, MA USA; 3 Massachusetts General Hospital, Boston, USA; 4 Harvard Medical School, Boston, USA; 5 AbbVie Pte Ltd, Singapore, Singapore; 6 Massachusetts Alzheimer’s Disease Research Center, Charlestown, USA

**Keywords:** Microglia, Alzheimer’s disease, Myeloid cells, Single-nucleus RNA-sequencing

## Abstract

Human microglia are critically involved in Alzheimer’s disease (AD) progression, as shown by genetic and molecular studies. However, their role in tau pathology progression in human brain has not been well described. Here, we characterized 32 human donors along progression of AD pathology, both in time—from early to late pathology—and in space—from entorhinal cortex (EC), inferior temporal gyrus (ITG), prefrontal cortex (PFC) to visual cortex (V2 and V1)—with biochemistry, immunohistochemistry, and single nuclei-RNA-sequencing, profiling a total of 337,512 brain myeloid cells, including microglia. While the majority of microglia are similar across brain regions, we identified a specific subset unique to EC which may contribute to the early tau pathology present in this region. We calculated conversion of microglia subtypes to diseased states and compared conversion patterns to those from AD animal models. Targeting genes implicated in this conversion, or their upstream/downstream pathways, could halt gene programs initiated by early tau progression. We used expression patterns of early tau progression to identify genes whose expression is reversed along spreading of spatial tau pathology (EC > ITG > PFC > V2 > V1) and identified their potential involvement in microglia subtype conversion to a diseased state. This study provides a data resource that builds on our knowledge of myeloid cell contribution to AD by defining the heterogeneity of microglia and brain macrophages during both temporal and regional pathology aspects of AD progression at an unprecedented resolution.

**Supplementary Information:**

The online version contains supplementary material available at 10.1007/s00401-024-02704-2.

## Introduction

Alzheimer’s disease is a progressive neurodegenerative disorder pathophysiologically characterized by depositions of amyloid-beta (Aβ) and abnormally phosphorylated tau (pTau) [23]. While Aβ deposits accrue relatively evenly throughout the neocortex, intraneuronal neurofibrillary tau pathology spreads in a stereotypical fashion from the entorhinal cortex (EC) to the hippocampus and the rest of the cortex, in stages defined as I–VI [4]. The rate of pTau accumulation correlates with the rate of cognitive decline [19]. Brain imaging technologies are quickly improving the ability to track pTau spreading in patients, and thus, pTau pathology is increasingly practical as a biomarker to identify intervention points for slowing cognitive decline.

Microglia, the myeloid cells of the brain, along with brain macrophages (perivascular, meningeal, and choroid plexus macrophages), have long been known to be involved in AD pathophysiology. Recent genetic evidence points toward a crucial contribution of these cells in disease susceptibility [25, 53]. As the primary phagocytes of the brain parenchyma, microglia may play a role in both clearance [31] and spreading of pTau aggregates [3, 22]. Indeed, targeting microglia reduces or prevents pathology in animal models [33, 47]. However, the precise biological role of microglia in human AD tau spreading remains unknown.

Single cell- and nuclei-RNA-sequencing (scRNA-seq/snRNA-seq) are powerful methods that have aided identification of various disease-associated microglia subpopulations in AD animal models [15, 27]. Recently, these approaches have been improved for the study of microglia in banked frozen human brains, by developing an enrichment protocol to capture much higher numbers of microglia, enabling the identification of numerically minor but disease relevant subpopulations [16]. However, many snRNA-seq AD studies have only included one or two brain regions (e.g., [34]) and/or only control and high pathology, but no intermediate pathology donors, thus sharply limiting characterization of microglial transcriptomic changes along AD-vulnerable neural networks and from early to late stages through intermediate stages.

We hypothesized that microglia transcriptomic changes parallel stereotypical spreading of pathological tau in the AD continuum, and that there is a distinct subpopulation of tau-responsive microglia with specific gene regulators that drive conversion from homeostatic microglia. To test this hypothesis, we isolated and analyzed single nuclei from 32 donors and 5 regions per donor across the Braak stages of tau pathology, from EC to primary visual cortex (V1). We confirmed canonical microglial marker expression across regions and pathology groups. Using multiple biochemical and histological readouts from the same tissue pieces used for snRNA-seq, we identified tau- and Aβ-pathology-associated microglia populations, including those involved in early and late pathology. Leveraging their spatial and temporal variability with respect to pathology, we further refined microglial signatures associated with tau and Aβ pathology, and investigated microglial subtype conversion to identify transitionally regulated genes, which are potential drivers of detrimental microglia states.

## Materials and methods

### Materials

#### Human tissue and donor selection

Thirty-two human donors were selected from the Massachusetts Alzheimer’s Disease Research Center (MADRC). Brain tissue was characterized according to established methods [39, 42]. Eight “Pathology Group 1” donors were selected based on the following criteria: (1) Primary neuropathological diagnosis of control (Not AD/low AD neuropathological changes burden) at postmortem examination by an MGH neuropathologist; (2) Braak neurofibrillary tangles (NFTs) stage 0-II as determined by the distribution of NFTs with a total tau immunostain and Bielchowsky’s silver stain [5]; (3) CERAD “C” plaque score of 0 [23]; and (4) the least possible concurrent pathologies (including α-synuclein and TDP-43). Eight “Pathology Group 2” donors were selected based on: (1) Primary, secondary, or tertiary neuropathological diagnosis of AD, (2) Braak neurofibrillary tangles (NFTs) stage II–III, (3) CERAD neuritic plaque score of 1–2 [23], and (4) the least possible concurrent pathologies. Eight “Pathology Group 3” donors were selected based on: (1) Primary neuropathological diagnosis of AD, (2) Braak NFT stage V; (3) CERAD neuritic plaque score of 2–3, and (4) the least possible concurrent pathologies. Eight “Pathology Group 4” donors were selected based on: (1) Primary neuropathological diagnosis of AD; (2) Braak NFT stage VI; (3) CERAD neuritic plaque score of 3, and (4) the least possible concurrent pathologies. Age of onset, age at death, postmortem interval, sex, *TREM2* R47H, and R62H mutations and *APOE* genotype were also collected (see below for detailed methods). All subjects or their next-of-kin provided written informed consent for the brain donation and the present study was approved under the MADRC Neuropathology Core Brain Bank Institutional Review Board.

Human brains were processed as described [12]. Briefly, all brains were separated into 2 hemispheres, one of which was postfixed in 10% formalin for 3 weeks. Regions of interest were embedded in paraffin following standard protocols [23, 39]. Four-micrometer-thick paraffin-embedded tissue sections were cut and placed on slides (Fisherbrand Superfrost Plus slides; Thermo Fisher Scientific) for histological analysis. The contralateral hemisphere was sliced coronally at the time of autopsy and 1 cm-thick slabs were flash frozen and stored at − 80 °C. Approximately 250 mg of tissue was dissected out of the frozen brain slab corresponding to Entorhinal cortex (EC), Posterior Parahippocampal Gyrus/Inferior Temporal Cortex (Brodmann Area 20; ITG), Dorsolateral Prefrontal Cortex (Brodmann Area 46; PFC), Visual Association Area (Brodmann Area 18/19; V2), and Primary Visual Cortex (Brodmann Area 17; V1), and kept at − 80 °C until processing for nuclei isolation and HT7/HT7 Tau aggregation assay. Approximately 10–25 mg of each brain region was dissected out of the frozen brain sections adjacent to the pieces taken for nuclei and kept at − 80 °C until homogenization for pTau/total Tau ELISA and HEK cell-based tau seeding assays.

### Methods

#### RIN screening for tissue selection

Approximately 10–20 mg of tissue from visual cortex was homogenized (Precellys CK14 beads), RNA was extracted (MagMAX mirVana Total RNA), and RNA Integrity Number (RIN) was measured on an Agilent 4200 Tapestation to select high-quality tissue for single nuclei-RNA-seq. RIN value was measured from 130 donors, for which 83 met the selected cutoff of RIN ≥ 5. Of these 83 donors, 32 were selected based on the criteria listed above. RIN values were additionally measured from the EC, BA20, BA46, V2, and V1 pieces used for snRNA-seq (mean ± SD: 5.3 ± 1.5).

### TREM2 R47H and R62H SNP genotyping

*TREM2* R47H (rs75932628) and R62H (rs143332484) single-nucleotide polymorphisms (SNPs) were genotyped using commercially available Taqman PCR assays on genomic DNA. Briefly, genomic DNA was purified from approximately 25 mg of frozen cerebellar cortex samples using the PureLink Genomic DNA Extraction Mini Kit (ThermoFisher Scientific, K182002), following the manufacturer's instructions. Next, DNA concentration was measured in a DS-11 spectrophotometer (DeNovix Inc) and 1.8 ng/μL working dilutions were prepared for the Taqman PCR assay. The reaction volume for the assay was 25 μL, comprising 1.25 μL of 20 × TaqMan *TREM2* R47H or R62H genotyping assay (ThermoFisher Scientific, Assay ID C_100657057_10 or C_172216876_10, respectively), 12.50 μL of 2 × TaqMan Fast Universal PCR Master Mix, no AmpErase UNG (Thermo Scientific, 4324018), and 11.25 μL of the DNA sample (20 ng). DNA samples were run in duplicates in 96-well plates (Bio-Rad) using a Bio-Rad CFX96 Touch Real-Time PCR Detection System. The amplification protocol involved an initial step at 95ºC for 10 min (ramp 1ºC/s), followed by 45 cycles of denaturation at 95ºC for 15 s and annealing/extension at 60ºC for 1 min. Allele discrimination and genotype assignment (CC, CT, or TT) were achieved through principal component analysis of VIC vs. FAM fluorescence, corresponding to base C (major allele) vs. T (minor allele), respectively. Minor alleles found in Taqman PCR assay indicating *TREM2* mutation were confirmed via PCR followed by amplicon sequencing. APOE genotype was identified by standard PCR-based restriction digestion or commercially available Taqman assays (ThermoFisher Scientific, cat#4351379, assays IDs: C___3084793_20 for rs429358 and C____904973_10 for rs7412) at MGH, or received from the National Centralized Repository for Alzheimer's Disease and Related Dementias (NCRAD).

### Nuclei isolation

Nuclei isolation was performed as described [16] with minor modifications. Briefly, fresh frozen tissue was cryosectioned (approximately 40 sections of 40 µm thickness) and lysed in sucrose lysis buffer [10 mM Tris HCl (pH 8.0); 320 mM sucrose; 5 mM CaCl_2_; 3 μM Mg(Ac)_2_; 0.1 mM EDTA; 1 mM dithiothreitol (DTT) and 0.1% Triton X-100]. Lysates were filtered through a 70 µm cell strainer. Nuclei were purified by ultracentrifugation (107,000 × *g* for 1.5 h at 4 °C) through a sucrose cushion (10 mM Tris HCl (pH 8.0); 1.8 M sucrose; 3 μM Mg(Ac)_2_; 0.1 mM EDTA and 1 mM DTT). Supernatants were removed and pellets were re-suspended in 2% BSA/PBS containing RNase inhibitor (0.2 U/μL) (Roche). Nuclei were incubated with fluorescently conjugated antibodies against the neuronal marker NEUN (RBFOX3/NEUN (1B7) AF647 mouse mAB, Novus Biologicals, NBP1-92693AF647) and the pan-oligodendrocyte/OPC transcription factor OLIG2 (Anti-OLIG2 clone 211F1.1 mouse mAb, Merck Millipore, MABN50A4). Samples were kept on ice throughout the isolation and staining procedure. Nuclei were stained with Sytox blue (Thermo Fisher) and sorted on a BD FACSAria Fusion. For each sample, we collected Sytox^pos^NeuN^pos^Olig2^neg^ and Sytox^pos^NeuN^neg^Olig2^neg^ (Fig. S1b).

### Pathological tau quantification

#### pTau231/Total tau

Tissue was homogenized (10–25 mg) in PBS containing protease (complete Mini #11836153001, Roche) and phosphatase inhibitors (phosSTOP #4906845001, Roche). Lysate was centrifuged for 10 min at 3000 × *g*, and supernatant was collected and used for ELISA and HEK cell tau seeding biosensor assay. Tau and phospho-Tau (Thr231) were measured from total brain lysate by MSD ELISA (MesoScaleDiscovery cat no. K15121) following the manufacturer’s protocol. Plates were developed using the MESO QuickPlex SQ 120 Plate Reader (MSD). Samples were run in triplicate and fit to an eight-point standard curve for total tau concentration determination.

#### HEK cell-based tau seeding assay

Tau bioactivity was measured as described [21], using human embryonic kidney (HEK) cells expressing a CFP/YFP FRET biosensor containing the tau repeat domain (ATCC, cat no. CRL-3275). Briefly, cells were cultured in 96-well plates to 60% confluency. Lysates were mixed with 1% lipofectamine 2000 in OPTI-MEM and 1 ug of total protein was added per well. After incubation for 14–18 h, cells were rinsed in PBS, trypsinized, and fixed with 4% paraformaldehyde. A Miltenyi VYB flow cytometer was used to measure mean FRET intensity and the percentage of FRET-positive cells per well. Multiplication of these values yielded the integrated FRET density (IFD). In addition, an AD positive control sample and a no pathology negative control sample were run on each plate and used to normalize values for comparisons across all samples. All samples were prepared in triplicate.

#### HT7–HT7 SIMOA

Single Molecule Array (SIMOA; Quanterix) bead-based tau aggregates assay was developed using a mouse anti-HT7 antibody (Thermo Fisher Scientific, RRID: AB_2314654) as both capture and detection. The assay was prepared according to the manufacturer’s protocol. Recombinant full length P301L tau aggregates were made as described [56] and were used as a calibrator and included in each run to generate standard curve. HD-X instrument, buffers, helper beads and streptavidin B-galactosidase, and enzyme substrate resorufin β-D-galactopyranoside were obtained from Quanterix. Assays were performed according to the manufacturer’s instructions. All samples were diluted in the Tau Calibrator Diluent (Quanterix).

### Immunohistochemistry

#### 3D6 Immunohistochemistry

Cryosections (10 µm thickness) were taken from the same pieces used for snRNA-seq. For Aβ immunohistochemistry, frozen cryostat sections adjacent to those used for snRNA-seq were subjected to immunohistochemistry with mouse monoclonal anti N-terminal Aβ antibody clone 3D6 (2 µg/mL).

#### Histological characterization of pathology and microglia

Paraffin-embedded tissue Sects. (4 µm thickness) were used for histological characterization of pathology and microglia markers. The tissue was stained with the following antibodies: mouse monoclonal anti N-terminal Aβ antibody clone 3D6 (1.2 µg/mL), rabbit monoclonal anti N-terminal Aβ antibody clone D54D2 (Cell Signaling 8243, 0.25 µg/mL), mouse monoclonal anti pTau antibody AT100 (Thermo Fisher MN1060, 0.006 µg/ml), mouse monoclonal anti CD68 antibody clone KP1 (Abcam ab955, 4 µg/mL), rabbit monoclonal anti C-terminal CD11c antibody clone EP1347Y (Abcam ab52632, 0.4 µg/mL), rabbit polyclonal anti TMEM119 antibody (Sigma-Aldrich HPA051870, 1 µg/mL), rabbit monoclonal anti CPM antibody clone EPR8052 (Abcam ab150405, 2 µg/mL), and rabbit monoclonal anti CD163 antibody clone EPR19518 (Abcam ab182422, 3 µg/mL).

Staining was performed on a Leica BOND Rx automated stainer using DAB or alkaline phosphatase-based detection (Leica). Sections were scanned in a slide scanner (3DHistech, Pannoramic 250 or Pannoramic 1000) and area fraction (i.e., % area of tissue section occupied by 3D6-immunoreactive plaques) was measured using the HALO software (Indica Labs, Albuquerque, NM, USA).

### Library preparation and sequencing

Single-nucleus cDNA libraries were constructed using the 10 × Genomics Chromium Single Cell 3‘Reagents Kit V3. Samples were pooled and sequenced targeting at least 30k reads per cell on a NovaSeq2000 at Discovery Life Sciences. Twelve libraries were selected based on low read numbers and low fractions of reads in cells and re-sequenced.

### Data preprocessing

Raw data were preprocessed with 10X Genomics CellRanger v4 (https://support.10xgenomics.com/single-cell-gene-expression/software/pipelines/4.0/release-notes) with 10X Genomics ‘GRCh38-2020-A’ pre-mRNA reference. Resequenced samples were merged at fastq level. Nuclei were quality checked and filtered to have exonic read counts > 100, mitochondrial gene percentages < 15% and at least 800 genes and UMIs per cell. Additional sample specific filtering was applied to remove potential outliers or low-quality cells by including only nuclei within range of log (median ± 3*MAD of number of genes/UMIs per cell) per sample. Samples were integrated across donors with Seurat rPCA integration [48] based on top 30 principal components, and brain myeloid cells were subsetted per brain region based on the following marker genes: *P2RY12, P2RY13, ITGAM, PTPRC, CX3CR1, SPI1, C1QA, C1QB, and TMEM119*. This resulted in 34, 28, 27, 28, and 24% of all nuclei per brain region, for EC, ITG, PFC, V2, and V1, respectively. Raw data corresponding to brain myeloid cell barcodes were then subsetted and Seurat CCA integrated [48] (v3.2.2) across donors for downstream analyses, based on top 30 principal components. Default Seurat parameters were used for shared nearest neighbor graph construction and Louvain clustering, with downstream analyses performed at clustering resolution 0.2, resulting in 14, 10, 11, 11, and 12 clusters per region, respectively.

### Public data QC comparison

For comparison of data quality against public studies, a number of donors and cells were extracted from respective publications [16]. Median UMIs [34] were extracted by downloading filtered read counts from synapse (https://www.synapse.org/#!Synapse:syn18485175) and subsetting to microglia based on ‘broad cell type’ annotation. Median UMI counts from [17] were taken from supplementary information, and median UMI counts were provided by the authors [16].

### Cross-region analysis

For cross-region analysis, region-specific data objects were randomly subsampled to 1000 cells per cluster to retain microglia heterogeneity. Subsamplings were further integrated across brain regions with Seurat CCA integration based on top 30 principal components and processed with default Seurat parameters. To confirm no sampling effect on downstream results, 10 different seeds were used, and downstream data objects compared. As results were highly similar, only results from a randomly selected subsampling are shown here. For comparison of EC-enriched microglia, the cross-region data object was clustered at resolution 3.1, and differential gene expression of cluster 4 vs. either all microglia across regions (Fig. 2c) or all other EC-region microglia (Fig. S2d) was assessed using the FindMarkers function of Seurat (v4.0.5, [20]), followed by a Reactome pathway enrichment analysis with ClusterProfiler (v4.2.2, [55]). Differentially expressed genes per region were then calculated using MAST [14], adjusting for donor ID as latent variable. These were further filtered for microglial markers determined from differential gene expression in a reference data set [16] comparing microglia to other cell types, with results filtered for adjusted *p* value < 0.05 and avg2logFC > 0.5. The top 5 microglial markers per region were visualized per dot plot. Cluster annotations used in region-specific brain myeloid data sets were in addition mapped to the cross-region data object to prove cross-region similarity of region-specific clusters (Fig. S3b). Clustering of the cross-region integrated data at resolution 0.4 resulted in 15 clusters (Fig. 3b). Differential gene expression per cluster was calculated using MAST [14], adjusting for donor ID and brain region as latent variables. Reactome pathway enrichment per cluster was calculated based on genes differentially expressed in comparison to homeostatic microglia (cluster 0) with logFC > 0.25, using all genes of the data object as background (Fig. S3c, Fig. S3d). Cross-region integrated brain myeloid cells were then binned into five equally sized classes of pTau/Total tau, HT7 aggregated tau, 3D6 IHC and HEK seeding readouts (low, low–medium, medium, medium–high, high) and their density was compared in a bin-to-bin pairwise fashion (e.g., low vs. low–medium, low–medium vs. medium, etc.). Bins with contributions of < 3 donors are not shown. Differential gene expression between bins was calculated using MAST, with donor ID and brain region as latent variables.

### Cross-region comparisons

The number of up- and downregulated genes differentially detected per region vs. all detected genes (across regions) was assessed (Fig. S2b. Spearman correlation analysis of aggregated expression across individual clusters and regions was performed, excluding donor-specific clusters, defined as those showing > 75% of individual donor contribution per cluster (Fig S2c). In addition, the overlap of detected genes (> 0 UMI counts in > 0.1% of brain myeloid nuclei per region) across regions was assessed (Fig. S2c).

### Region-specific analysis

Per region, clusters were tested against expected proportions of pathology groups using binomial tests, and significant enrichment of pathology groups over expected proportions is indicated (*) at adjusted *p *value < 0.001 and enrichment of >  = 10%. Spearman correlation of normalized microglia proportion per cluster with tau and Aβ readouts was assessed and considered significant at nominal *p* value < 0.05 (Fig. 3a). Differential gene expression was calculated per cluster vs. each other cluster per region (Table S4), if at least 100 nuclei were present, and considered significant at adjusted *p* value < 0.01, average logFC > 0.2 and at least 10% of cells expressed per subcluster. Reactome pathway enrichment analysis was calculated per cluster and region for the comparisons against HOM microglia (Table S5).

### Effect of concurrent pathologies and genotypes on microglial clustering

To assess the brain myeloid cell transcriptome changes as a function of co-existence of α-synuclein and TDP-43 pathology as measured in [39], the distribution of nuclei coming from samples with positive staining (*n* = 2 donors with α-synuclein pathology, *n* = 7 donors with TDP-43 pathology) was visualized in the cross-region and region-specific datasets. Both TDP-43 and α-synuclein pathology-specific nuclei were well distributed across clusters that were not either donor-specific or -enriched. To test this further, proportional analysis was performed, statistically assessing the change of proportion of TDP-43 positive versus TDP-43 negative nuclei per cluster and of α-synuclein positive versus α-synuclein negative nuclei per cluster. No significant differences between groups in any cluster for both of these analyses were found after multiple testing adjustment, indicating that the pathology co-existence did not affect individuals’ myeloid phenotypes or populations. An identical approach was taken for *APOE* genotypes and TREM2 variant carriers (one donor was found to carry the R62H variant and two donors the R47H variant). *APOE* genotypes and *TREM2* variant carrier nuclei were well distributed across clusters that were not donor-specific or-enriched, and proportional analysis statistically assessing (i) the change of proportion between *APOEƐ*3/Ɛ3 and *APOEƐ*3/Ɛ4 (genotypes with largest sample sizes), (ii) the change of proportion of *TREM2* variant carriers versus non-carriers per cluster, and (iii) the change of proportion of R47H carriers versus non R47H carriers per cluster resulted in no significant differences between groups in any cluster for all three of these analyses after multiple testing adjustment, indicating that in our data, *APOE* genotypes and *TREM2* variant carriers are not outliers and do not cause development of myeloid cells of individual phenotypes or populations. While the integration applied across donors might contribute to masking genotype-specific myeloid phenotypes, sample sizes of individual concurrent pathologies and genotypes of interest (i.e., *APOEƐ*4 homozygotes and *TREM2* variants) were too small for an alternative genotype/pathology-specific analysis strategy.

### Public genelist comparison

Comparison of cluster markers in comparison to HOM cluster 0 from cross-region integrated brain myeloid cells, and per region brain myeloid data, respectively (Fig. 3d, Fig. S3a), with genelists from public data included i) AD1 and AD2 signatures from [16], ii) laser capture microdissected samples from [9], with the following signatures:”Das_LCM_Plaque” (ThioflavinS + plaques), “Das_LCM_Peri_Plaque” (50µm area around plaques), “Das_LCM_NFT” (neurofibrillary tangles with the 50 µm area around them), “Das_LCM_Distant” (area > 50 µm away from plaques), “Das_LCM_Plaque_vs._NFT” (ThioflavinS + plaques vs. neurofibrillary tangles), iii) CRM2 (cytokine response 2), CYT/CRM1 (cytokine response 1), DAM (disease-associated), HLA (antigen-presenting response), HM (homeostatic), IRM (Interferon response), RM (ribosomal response), TRANS (transitioning CRM) signatures from [32], and iv) tau fibril response genes from [51]. For comparison with mouse genelists, mouse gene symbols were converted to human gene symbols with biomaRt (v2.50.3, [13]). Differentially expressed genes from sc/snRNA-seq studies were filtered to include those detected in at least 5% of cells/nuclei per comparison group, with adjusted *p* value < 0.05. Differentially expressed genes from bulk RNA-seq studies were filtered for genes with adjusted *p* value < 0.05. Differentially expressed genes from the laser capture bulk study were filtered for genes with nominal *p* value < 0.05, as done in the study. Significant Spearman correlation of genes is indicated by nominal *p* value, at a minimum number of 10 overlapping genes between studies.

### Trajectory analysis

Pseudotime was calculated for microglial trajectories to disease-associated clusters (HOM cluster to clusters 3 (DAM1), 4 and 5 of cross-region integrated data) with monocle3 [7]. After conversion to a CellDataSet, data were re-normalized based on top 30 PCs and aligned based on donor IDs using Batchelor [18]. The number of counts and mitochondrial gene percentage were regressed out using a linear model based on cells’ PCA coordinates. Leiden clustering was applied (at *k* = 20, resolution = 6e^−5^) and compared to Seurat clustering, resulting in similar patterns. The largest partition covering all brain myeloid cell phenotypes was subset and trajectories defined from homeostatic microglia individual disease-associated clusters. For each trajectory expression along pseudotime was aggregated into 100 bins and filtered to keep only genes detected in > 50 bins. Further, genes differentially expressed along pseudotime were determined using Moran’s *I* test, based on the principal graph, and filtered for (i) adjusted p value < 0.001, (ii) > 100 cells in which the gene was expressed, and (iii) Moran’s test statistic > 10. Among them, transitionally expressed genes along pseudotime were further identified by splitting the pseudotime into four quartiles and keeping genes showing a higher expression in middle quartiles compared to the first and last one. Expression was scaled per gene and visualized for each trajectory.

### Microglia subtype mapping

Comparison with previous mouse microglial signatures [27, 28] was performed based on averaged geneset expression per cluster (Fig. 4c, Fig. S4b). From Kim et al., the top 30 genes per microglial phenotype were used as signature; from Keren-Shaul et al., genes depicted in Fig. 6 were used.

### Pseudobulk analysis

Pseudobulk analysis was applied on sum aggregated expression levels across cells per sample (v1.4.0, [35]). edgeR (v3.36.0, [8]) differential gene expression (glmQLFit) was calculated to identify genes differentially expressed between regions (Fig. 5a). Results were adjusted for gender; RIN values were not significantly different between compared groups. Reported genes were filtered for genes not showing significant region differences in pathology group 1. For visualization, pseudogenes and non-coding protein genes were filtered out.

### Gene clustering

To identify gene expression patterns along brain regions decreasingly affected by tau pathology (EC > ITG > PFC > V2 > V1), genes from cross-region integrated microglia were subset per pathology group, sum aggregated per region, z-score-normalized, and k-means clustered (Fig. 5b). The cluster number was determined by Elbow plot across a range of k of 1 to 10, with 15 maximum iterations, and 50 random sets. Sankey diagrams were added using networkD3 (v0.4, https://cran.r-project.org/web/packages/networkD3/index.html). Reactome pathway enrichment was calculated with clusterProfiler (v4.2.2, [55]) using all detected genes in the cross-region integrated data as background (Fig. S3f). To determine genes strongly correlated with a given pathology, Spearman correlation per gene cluster and pathology group was performed against the different biochemical readouts.

## Results

### A large single-nucleus RNA-seq atlas to study transcriptomic changes of brain myeloid cells along the spatiotemporal progression of AD

**Fig. 1:**
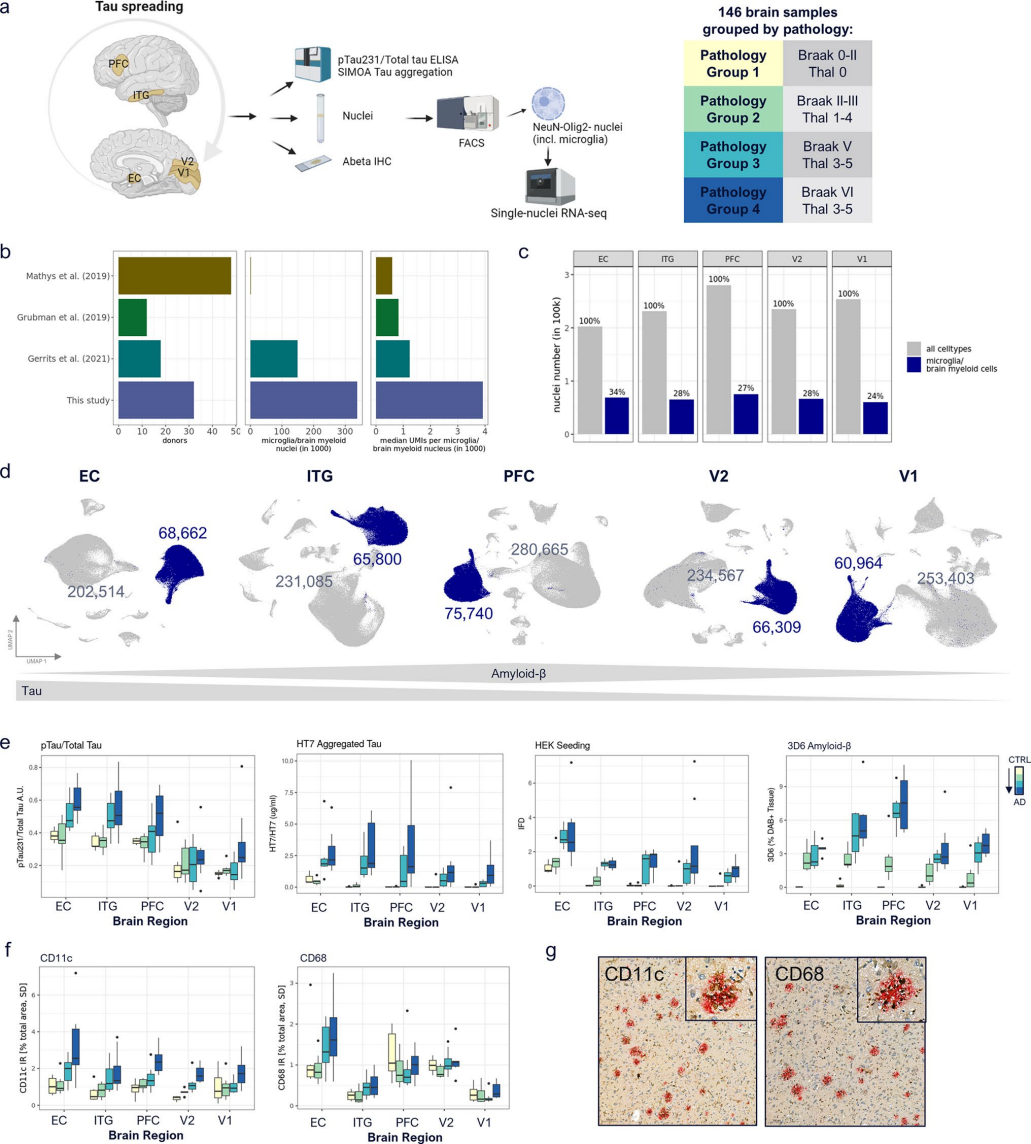
Study design and identification of brain myeloid cells across brain regions. **a** Study design. Samples from 5 brain regions of in total 32 donors along four stages of AD pathology progression were snRNA-seq profiled and characterized by quantitative readouts of tau as well as Aβ 3D6 IHC. Samples were divided into 4 pathology groups, according to their Braak and Thal stage. **b** Comparison of dataset size and median UMIs per microglia/brain myeloid nucleus vs. public microglial studies. **c** Per region microglia/brain myeloid cell numbers as proportion of all NeuN-/Olig2- cells per region. **d** UMAP representation of NeuN-/Olig2- cells, brain myeloid cells are colored in blue. Grey and blue numbers correspond to the absolute NeuN-/Olig2- sorted non-myeloid cells (e.g., astrocytes, endothelial cells, and pericytes) and myeloid cells, respectively. **e** pTau/Total Tau, HT7 Aggregated Tau, HEK seeding, and 3D6 Amyloid-β measurements for each pathology group (CTRL → AD), across brain regions. **f** Quantification of CD11c and CD68 immunohistochemistry across brain regions and pathology groups. **g** Representative CD11c and CD68 IHC (EC, grey matter) of pathology group 4 samples, with CD11c in brown and plaques (3D6) in red, and CD68 in brown and plaques (D54D2) in red, respectively (scale bar 100 µm). IHC across pathology groups in Fig S1d/e

Brain tissue samples from 32 donors at varying stages of tau pathology were split into 4 groups based on prior neuropathological characterization (Fig. S1a, Table S1). To capture myeloid cells along the stereotypical progression of tau pathology, we selected 5 brain regions that included allocortex and neocortex, from expected high to low pathology: entorhinal cortex (EC), inferior temporal gyrus (ITG), prefrontal cortex (PFC), visual association cortex (V2), and primary visual cortex (V1) (Fig. 1a). Compared to pioneering snRNA-seq studies [17, 34], we captured more than 150 times the number of brain myeloid cell nuclei per tissue sample with our enrichment protocol (337,475 total). Further, separation of nuclei by their corresponding cell types combined with deeper sequencing led to increased numbers of genes (more than three times the number of median UMIs) detected per myeloid cell nucleus compared to any published study (Fig. 1b). With our enrichment protocol (Fig. S1b), brain myeloid cells amounted to 24–34% of total nuclei per region (Fig. 1c, d).

### Biochemical and neuropathological characterization reveals brain myeloid cell responses associated with spatiotemporal aspects of AD pathology

To correlate transcriptomic changes in myeloid cells with local levels of pTau and Aβ pathology, we conducted an extensive biochemical and immunohistochemical quantitative analysis in samples adjacent to those used for snRNA-seq. Tau protein becomes hyperphosphorylated early in disease, which contributes to its aggregation [2]. In line with prior histological and biochemical studies (e.g., [11]), we observed a pattern of pTau/Tau levels of EC > ITG > PFC > V2 > V1 (Fig. 1e, Fig. S1c). In a given brain region pTau/Tau levels reflected the pathology groups, with donors of pathology group 4 (dark blue, Braak VI) and 1 (yellow, Braak 0/I/II) showing highest and lowest levels of pTau, respectively. A similar pattern was seen for HT7 aggregated tau and the propensity of lysate material for tau seeding by HEK biosensor cells (HEK seeding). As expected, Aβ pathology, as measured by 3D6 immunoreactivity, was highest in neocortex (PFC and ITG) (Fig. 1e). These pathology readouts confirmed earlier studies and revealed the expected pathology levels in the brain samples selected for this study.

To determine the dynamics of reactive microglia with respect to disease progression, we stained FFPE tissue sections from the contralateral hemisphere to that used for snRNA-seq with antibodies for reactive microglia (CD11c and CD68) and plaques (3D6), and quantified the area covered by microglia markers, plaques, and co-localized area. CD11c (encoded by *ITGAX*) increased from pathology group 1 to pathology group 4, and showed highest expression in EC (high-tau) and PFC (high-tau & Aβ), suggesting first a tau-associated (path. group 3) and later tau & Aβ associated (path. group 4) changes (Fig. 1f, g, Fig. S1d). On the other hand, CD68 protein expression increased from pathology group 1 to group 4 in EC, ITG, and V1, and had highest immunoreactivity in EC followed by PFC within all pathology groups, suggesting an association with early tau and early Aβ pathology (Fig. 1f, g, Fig. S1e). Thus, these typical reactive microglia markers are both associated with tau and Aβ pathology progression, but demonstrate unique spatial and temporal patterns.

### Comparison of myeloid cells across brain regions reveals an EC-specific signature

**Fig. 2:**
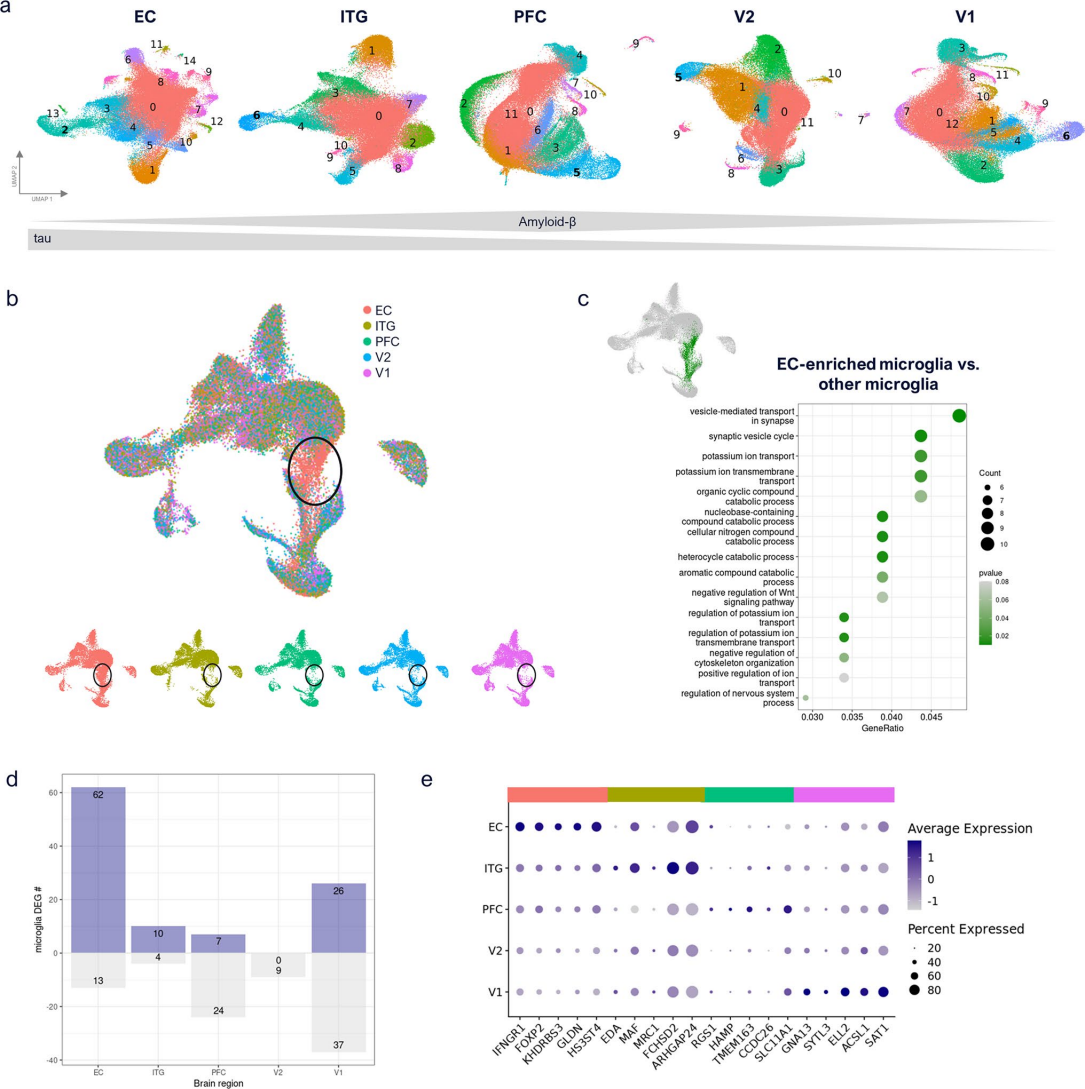
Brain myeloid cell similarity across brain regions. **a** Brain myeloid cell subclustering per brain region. Macrophage cluster numbers are indicated in bold (based on *LYVE1*, *MRC1*, *CD163,* and *F13A1* marker genes). **b** Cross-region integration of subsampled brain myeloid cells across brain regions shows alignment between regions for most cells, except for one EC-enriched population of cells (highlighted in black circles). Shown are combined and per region UMAP plots. **c** EC enriched population (indicated as green cells in UMAP plot) was compared to all other brain myeloid cells across regions. Biological process GO term enrichment indicates upregulated synapse vesicle cycle changes and ion transport differences. **d** Up- and downregulated differentially expressed gene (DEG) numbers per region, filtered for microglial genes (average log2FC > 0.25). **e** Top 5 upregulated microglia genes per region (no significantly upregulated V2 markers identified)

Clustering of brain myeloid cell nuclei based on their transcriptomes showed few donor-specific clusters after mapping across all donors (Fig. 2a, Fig. S2a). When integrating brain myeloid cells across different brain regions, these appeared to be very similar (Fig. 2b), with < 1% of detected genes being differentially regulated in any given region (Fig. S2b, Table S2) and with high correlation of clusters between regions (Fig. S2c, Table S3). Although most brain myeloid cells were highly similar across brain regions, one group of brain myeloid cells from EC clustered separately from those in other regions and showed differentially expressed genes (DEGs) associated with vesicles and potassium transport (Fig. 2c, Fig. S2d). Notably, this cluster was observed across all donors, and was neither specific to donors with high or low pathology, nor enriched for Aβ or tau pathology readouts (Fig. S2e, f). Although the relative proportion of DEGs in any given region was small, EC also showed the most DEGs of any of the 5 regions [75 genes, 62 up—including *IFNGR1* (encoding the interferon gamma receptor 1)—and 13 down], followed by V1 (67 DEGs, 26 up and 37 down), while V2 showed the least DEGs (zero genes) (Fig. 2d, e). In summary, this analysis revealed a unique transcriptomic signature of myeloid cells in EC, highlighting allocortical vs. neocortical differences that might contribute to differences in vulnerability to tau.

### Correlation with global and local AD neuropathology reveals distinct homeostatic and AD-associated brain myeloid states

To identify brain myeloid cell subsets associated with AD pathology, we analyzed the percentage of myeloid cell nuclei per cluster from high and low-pathology donors, including microglia and perivascular macrophages (PvMs) as identified by marker genes *LYVE1, MRC1, F13A1,* and *CD163*. We reasoned that AD-associated brain myeloid cell clusters should have a significantly higher proportion of nuclei from high vs. low-pathology donors and/or correlate positively with any of the local tau and Aβ pathology readouts. By contrast, homeostatic microglia clusters should have a higher proportion of nuclei from low vs. high pathology donors and/or correlate negatively with the local tau and Aβ pathology readouts.

**Fig. 3:**
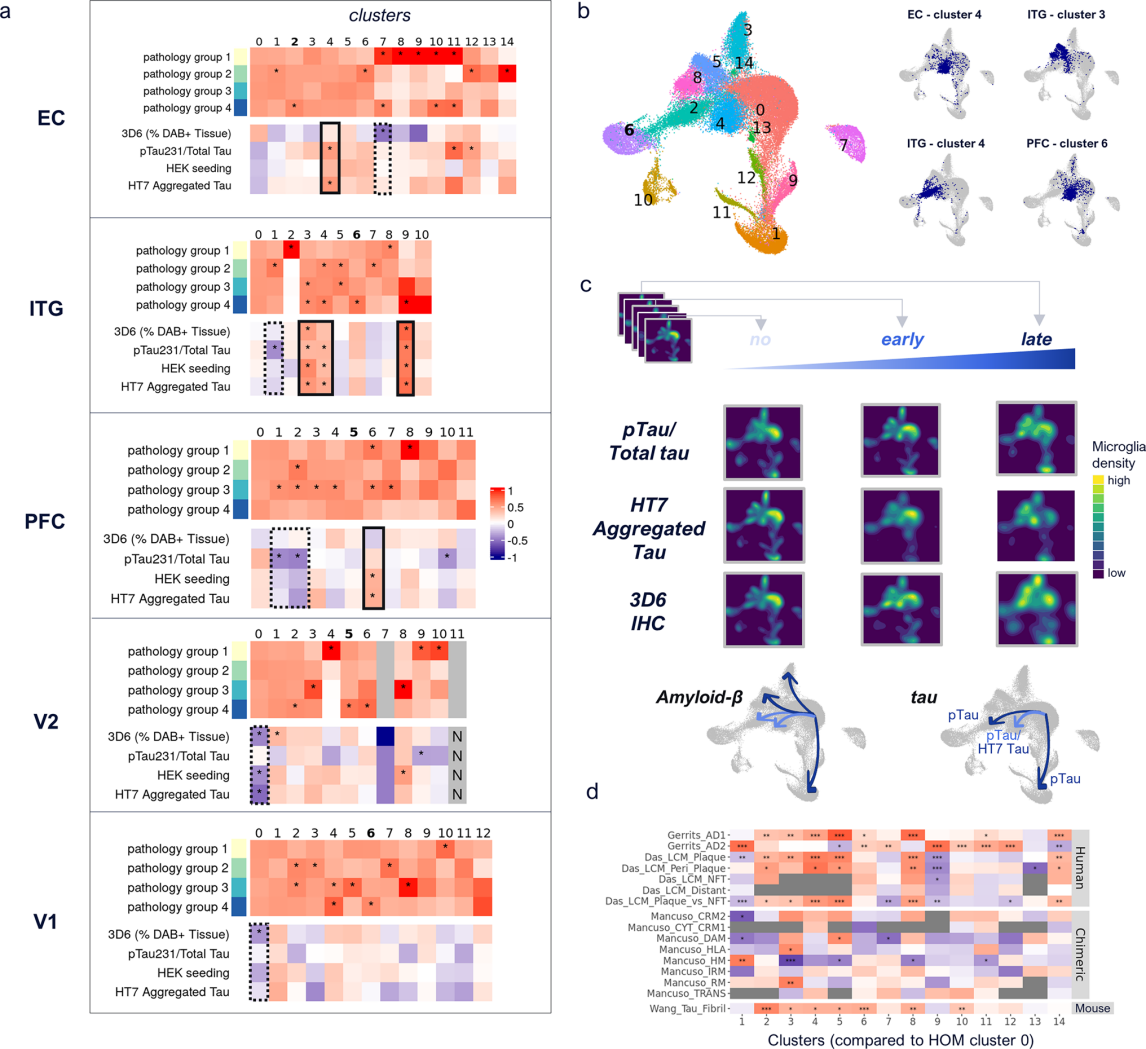
Identification of tau- and Aβ -associated microglia and brain macrophage subpopulations. **a** Per cluster pathology group enrichment shown as observed over expected ratios (scaled to 1) (upper panels of heatmaps) and Spearman correlation of 3D6 and tau readouts with proportion of brain myeloid cells per cluster (lower panels of heatmaps). ‘*’corresponds to significant enrichment >  = 10% (binomial test, adj. *p* value < 0.001), and significant Spearman correlation (*p* value < 0.05), respectively. Solid black boxes denote clusters positively correlated with pathology; dashed black boxes denote clusters negatively correlated with pathology. Bold cluster numbers indicate macrophage clusters, characterized by increased expression of *LYVE1*, *MRC1*, *F13A1*, and *CD163*. **b** Mapping of disease-associated clusters per region (right) to cross-region integrated data (left) confirms similarity of disease-associated clusters across brain regions, albeit indicating expression differences between primarily tau- (clusters 2/4 in integrated data) and tau + Aβ -associated clusters (clusters 5/8 in integrated data). **c** 3D6 IHC, pTau/Total tau, and HT7 aggregated tau readouts were binned into 5 equally spaced categories, representing no-to-late pathology. For simplicity, integrated microglia are shown for no, early, and late pathology only (first, middle, last bin), based on their cellular density in individual clusters (UMAP representation). Grey plots beneath visually summarize shifts of brain myeloid cells into clusters stratified for early (lightblue) and late (darkblue) pathology. For HT7 aggregated tau, bin #4 (not #5) is shown at late stage, as last bin (#5) only contained data from one donor. **d** Spearman correlation of cross-region brain myeloid cell clusters (using DEGs per cluster vs. cluster 0) with public genelists. Significant correlation indicated by ***p value < 0.001, **p value < 0.01, *p value < 0.05, grey boxes indicate insufficient data (number of overlapping genes between data sets < 10). AD1 and AD2 human microglia signatures from [16]; laser capture microdissected samples from [9] with signatures “Das_LCM_Plaque” (ThioflavinS + plaques), “Das_LCM_Peri_Plaque” (50 µm area around plaques), “Das_LCM_NFT” (neurofibrillary tangles with the 50µm area around them), “Das_LCM_Distant” (area > 50µm away from plaques), “Das_LCM_Plaque_vs_NFT” (ThioflavinS + plaques vs. neurofibrillary tangles); human iPSC-derived microglia-like cells transplanted into mice, with signatures *CRM2* cytokine response 2, *CYT/CRM1* cytokine response 1, *DAM* (disease associated), *HLA* antigen-presenting response, *HM* homeostatic, *IRM* (Interferon response), *RM* (ribosomal response), *TRANS* transitioning CRM from [32]; and primary mouse microglia tau fibril response genes from [51]

Screening for AD-associated microglia, we detected several clusters with a significantly higher number of high- and low-pathology donor myeloid cell nuclei than expected by chance. For example, ITG microglia clusters 3 and 4 showed significantly more high pathology (pathology group 3 and/or 4) donor nuclei (Fig. 3a, ITG upper heatmap; cluster 3: adj. *p* values 5.3e-7 and 8.5e-8, respectively; cluster 4: adj. *p* value 2.2e-14). Further, we identified several clusters for each brain region for which the percentage of nuclei per donor was correlated with its pathology readout. For example, the proportion of microglia in ITG cluster 3 showed a significant positive correlation with all tau and Aβ pathology readouts (Fig. 3a, ITG lower heatmap; 3D6 *p* value < 0.01, pTau231/Total Tau *p* value < 0.05, HEK seeding *p* value < 0.001, HT7 Aggregated Tau p value < 0.01). Importantly, the gene signature of this cluster (i.e., DEGs as compared to cluster 0, which was equally contributed by all four pathology groups and did not correlate with any pathology readout) also positively correlated with the “AD1” human AD microglia described by Gerrits et al. [16] and negatively correlated with human homeostatic microglia reported by Mancuso et al. [32] (Fig. S3a, ITG heatmap, “Gerrits_AD1” *p* value < 2.22e-16, “Mancuso_HM” p value 1.67e-10), reinforcing the identity of this microglia cluster as the AD-associated microglia. Overall, microglia clusters that positively correlated with tau or with tau & Aβ pathology were mainly observed in early tau regions (e.g., EC cluster 4, ITG clusters 3, 4 and 9) (Fig. 3a, solid black boxes), and these clusters were characterized by genes in pathways including “Scavenging by Class A Receptors” (EC cluster 4, ITG clusters 3 and 4), and “Cell recruitment (pro-inflammatory response)” (ITG cluster 3) (Table S4, Table S5). The proportion of PvM clusters did not show any significant correlations with any of the pathology readouts, but did show increased proportion of nuclei from pathology group 4 donors in EC, ITG, V2, and V1 (proportions EC—7.3%; ITG—3.1%; PFC—7.2%; V2—3.2%; V1—1.4%; adj. *p* values EC 4.7e-15, ITG 2.2e-14, V2 5.4e-12, and V1 4.77e-5, respectively).

Regarding homeostatic microglia, microglia clusters negatively correlated with pathology were mainly observed in later tau regions, as expected (V2 cluster 0: 3D6 p value < 0.01, HEK Seeding *p* value < 0.05, HT7 Aggregated Tau *p* value < 0.01; V1 cluster 0: 3D6 *p *value < 0.05) (Fig. 3a, dashed black boxes). These microglia clusters showed typical markers of microglia homeostasis, e.g., *P2RY12*, and newly identified homeostatic microglia genes, such as *SYNDIG1, FOXP2, OXR1*, and *LINC02232* (Table S4)*.* Among the microglia clusters negatively correlated with pathology were ITG cluster 1 and PFC cluster 2 (Fig. 3a, dashed black boxes). Both showed a significant negative correlation with the pTau/total Tau ratio (Fig. 3a; *p* values < 0.05 and 0.01, respectively) and were characterized by an increased expression of ribosomal genes associated with translation and viral transcription, as well as iron uptake and storage genes *FTL* and *FTH1*, encoding ferritin protein light and heavy chains, respectively (Table S5). Thus, in this study, *FTL* + microglia did not increase with pTau or Aβ load, unlike previously reported [26], but rather showed significantly decreased proportions with increasing tau progression. Furthermore, ferritin-positive microglia have previously been described as “dystrophic” and “senescent” (e.g., [30]); however, we did not observe any enrichment of genes or pathways associated with apoptosis or senescence within these clusters (Table S5). These microglia clusters (ITG cluster 1 and PFC cluster 2) showed a high similarity to ribosomal response microglia recently described in a human microglia transplantation model [32] (Fig. S3a, “Mancuso_RM”).

To confirm the observed microglia and PvM phenotypes at the protein level and their localization with respect to pathology, we performed immunohistochemistry in the ITG region across all donors using antibodies for markers of pathology-associated (CPM) and homeostatic (TMEM119) microglia, and PvMs (CD163), with nearly adjacent sections (between 30 and 100 µm away) stained for amyloid plaque and tau pathology (Fig. S3b-e). We did not observe a difference in TMEM119 immunoreactivity between pathology groups, suggesting that homeostatic microglia are not correlated with pathology. However, we did observe an increase in CPM immunoreactivity with respect to pathology groups, and CPM positive cells were observed adjacent to plaques and dystrophic neurites, according to nearly adjacent sections stained with 3D6 and AT100, respectively. These results are in line with the observed transcriptional changes: *TMEM119* is a homeostatic marker of ITG cluster 0, whose proportions are not significantly different with respect to pathology group or pathology readouts. *CPM* is a marker of ITG cluster 3, whose proportion was significantly positively associated with pathology group 3 and 4 donors as well as with all 4 pathology readouts in ITG. CD163 immunoreactivity was observed in cells with a monocyte/ macrophage-like morphology in the brain vasculature (Fig. S3d, arrow). Total levels of CD163 immunoreactivity did not change with respect to pathology, with no significant differences observed between pathology groups. This trend is in line with our observation of increased *CD163* gene expression in cluster 6 of ITG, which matches the transcriptomic profile of PvMs and does not show any correlation with pathology groups. We also observed scattered CD163-positive cells in the brain parenchyma (Fig. S3d, arrowhead), and an increase in this parenchymal CD163 in 2 donors of pathology group 4, a finding which would need to be confirmed in a larger cohort. Interestingly, ITG microglia cluster 4 showed significantly increased *CD163* as compared to all other clusters, and a significant correlation with higher pathology groups. This cluster did not have an overtly PvM-like phenotype based on transcriptomic profile, suggesting that it may align with the CD163-positive parenchymal microglia-like cells that we observed by IHC that increased in 2 pathology group 4 donors. The parenchymal CD163-positive cells were not abundant enough to determine the exact localization with respect to amyloid or tau pathology in nearly adjacent sections in these 2 donors, despite their trend toward increase in relation to overall pathology load.

In summary, correlations between local biochemical and neuropathological measures of tau and Aβ pathology and microglia transcriptomic clusters enabled us to discern between homeostatic and AD-associated microglia in multiple brain regions.

### Correlation with local tau vs. Aβ measures reveals distinct subsets of AD-associated microglia

Once established that the existence of AD-associated microglia is distinct from homeostatic microglia, we aimed to identify associations between microglia clusters and local tau vs. Aβ measures that may indicate specialized responses to one or the other pathology. Prior research suggested that microglia show unique responses to tau vs. tau & Aβ [16]. Thus, we investigated marker genes and pathways in microglia clusters positively or negatively correlated with tau measures, but with no significant association to Aβ pathology, like EC cluster 4 and PFC cluster 6. EC cluster 4 showed upregulated markers of hypoxia and inflammatory response (*HIF1A, DUSP1, FOS*) and was represented by pathways including “response to decreased oxygen levels” (Table S5). Moreover, it correlated with cytokine response (“Mancuso_CYT_CRM1” *p* value 3.33e-3, “Mancuso_CRM2” *p* = 1.26e-4), HLA (“Mancuso_HLA” *p* = 2.75e-3), and tau fibril-treated microglia (“Wang_Tau_Fibril” *p* = 2.81e-6) as identified in published studies (Fig. S3a). PFC cluster 6 showed markers and pathways similar to those of EC cluster 4, including “response to decreased oxygen levels”, and exhibited a positive correlation with cytokine response microglia identified by Mancuso et al., 2022 (“Mancuso_CRM2” *p* = 3.43e-5) (Fig. S3a, Table S5).

Only V2 cluster 1 showed a significant positive correlation with Aβ (3D6 *p* < 0.05) but not tau, and only two clusters (EC cluster 7 and V1 cluster 0) had a significant negative correlation with Aβ (both 3D6 p values < 0.05) but not tau. Finally, ITG clusters 3 and 9 correlated positively and V1 cluster 0 correlated negatively with both Aβ and tau readouts (ITG cluster 3: *p* values see above; ITG cluster 9: 3D6 *p* < 2e-5, pTau231/Total Tau *p* < 0.001, HEK seeding *p* < 0.001, HT7 Aggregated Tau *p* < 5.53–5; V1 cluster 0: 3D6 *p* < 0.05). While several tau and tau & Aβ-associated clusters showed significant correlation with Gerrits et al. “AD1” tau & Aβ signature (e.g., Fig. S3a, ITG microglia clusters 3 and 9, *p* values < 2.22e-16 and < 2.3e-6, respectively), none of the tau-only associated clusters (e.g., EC cluster 4, ITG cluster 4, PFC cluster 6) showed a positive correlation with Gerrits et al. “AD2” tau-only signature, based on fold change comparisons against homeostatic microglia (Fig. S3a).

These data suggest that there are distinct transcriptomic responses of AD-associated microglia to tau vs. Aβ pathology as well as a signature common to both pathologies. To better understand the similarities and differences in homeostatic, tau and Aβ-associated clusters between regions, we mapped the homeostatic and pathology-associated clusters from individual regions to our cross-region clusters. Individual region microglia clusters showing a negative correlation with tau pathology (Fig. 3a, Fig. S3a, S3f, dashed boxes/ circles) aligned into cross-region clusters 0 and 3 (Fig. 3b, Fig. S3f). Moreover, AD pathology-associated EC cluster 4 and PFC cluster 6 aligned with cross-region cluster 4, while ITG clusters 3 and 4 mapped to cross-region clusters 5, 8, and 2, respectively (Fig. 3b, S3f). Genes characterizing these cross-region pathology-associated clusters 2, 4, 5, and 8 include the top regulated genes *CD163* (a typical marker of brain macrophages) and *RGS1, PTPRG,* and *CPM*, respectively (Fig. S3g, Table S6, Fig. S3h, Tables S7), and pathways such as “CDC42 GTPase cycle”, and “binding and uptake of ligands by scavenger receptors” (Fig. S3i, Table S8). PvMs (*CD163, LYVE1, MRC1, and F13A1)* were mainly found in cross-region cluster 6, while cluster 10 was marked by increased expression of *CCR2*, suggesting that cells in this cluster constitute myeloid cells of a peripheral origin, e.g., monocytes (Fig. S3j).

### Identification of shifts in microglia states from homeostatic to AD-associated

Next, we asked whether homeostatic microglia transition to an AD-associated state along the disease course. We first investigated shifts in microglial density from homeostatic to AD-associated along the accrual of pTau/Total tau, HT7 aggregated tau, and Aβ plaques. We binned these readouts into 5 classes and plotted the density of microglia in each pathology bin and cross-region cluster (Fig. 3c; 3 of 5 bins [no/early/late] shown for simplicity). Interestingly, of the cross-region AD-associated clusters 2, 4, 5, and 8, clusters 2 and 4 showed the highest density of microglia nuclei in the early and late tau bins, while clusters 5 and 8 showed a high density of microglia nuclei in the late Aβ bin (Fig. 3c, Table S9, Table S10), suggesting differential early vs. late responses of these microglial clusters to tau and Aβ pathologies. Notably, the tau progression-associated clusters 2 and 4 showed correlations with Gerrits et al. “AD1” and tau fibril-treated microglia (Fig. 3d, Gerrits et al. “AD1” *p *values < 0.01 and < 2e-5, respectively, and “Wang_Tau_Fibril” *p* values < 2.8e-6 and < 0.05, respectively). Furthermore, the Aβ-associated cross-region clusters 5 and 8 showed the strongest correlation with “AD1” signatures (rho = 0.8 and 0.7, respectively; *p* values < 2.22e-16 for both), positive correlations with laser capture microdissected plaques and peri-plaque signatures (“Das_LCM_Plaque”: rho = 0.61 and 0.55, with *p* < 8.3e-6 and < 1.9e-6, respectively, and “Das_LCM_Peri_Plaque”: rho = 0.54 and 0.56, with *p* < 0.05 and < 0.01, respectively) [9], and a negative correlation with “Mancuso_HM” (homeostatic microglia, rho =  – 0.48 and – 0.49, with both *p* values < 0.05), and cluster 5 additionally showed a positive correlation with “Mancuso_DAM” (*p* < 0.05) (Fig. 3d). Interestingly, the tau-associated cross-region clusters 2 and 4 did not show significant positive correlations with any of the Mancuso et al. signatures or the Gerrits et al. “AD2” tau signature (Fig. 2d), but cluster 4 did show positive correlations with “Das_LCM_Plaque”, “Das_LCM_Peri_Plaque”, and “Das_LCM_Plaque_vs_NFT” (*p* < 2.13–5, < 0.05 and *p* < 0.001, respectively). This suggests that the Gerrits et al.’s study, with limited brain regions, and the Mancuso et al.’s study, which used human iPSC-derived microglia transplanted into mouse brain, incompletely describe the microglia and macrophage signatures that we were able to detect in human AD across all 5 brain regions.

### In silico modeling identifies “phasic” genes as potential regulators of microglia transition during disease

**Fig. 4:**
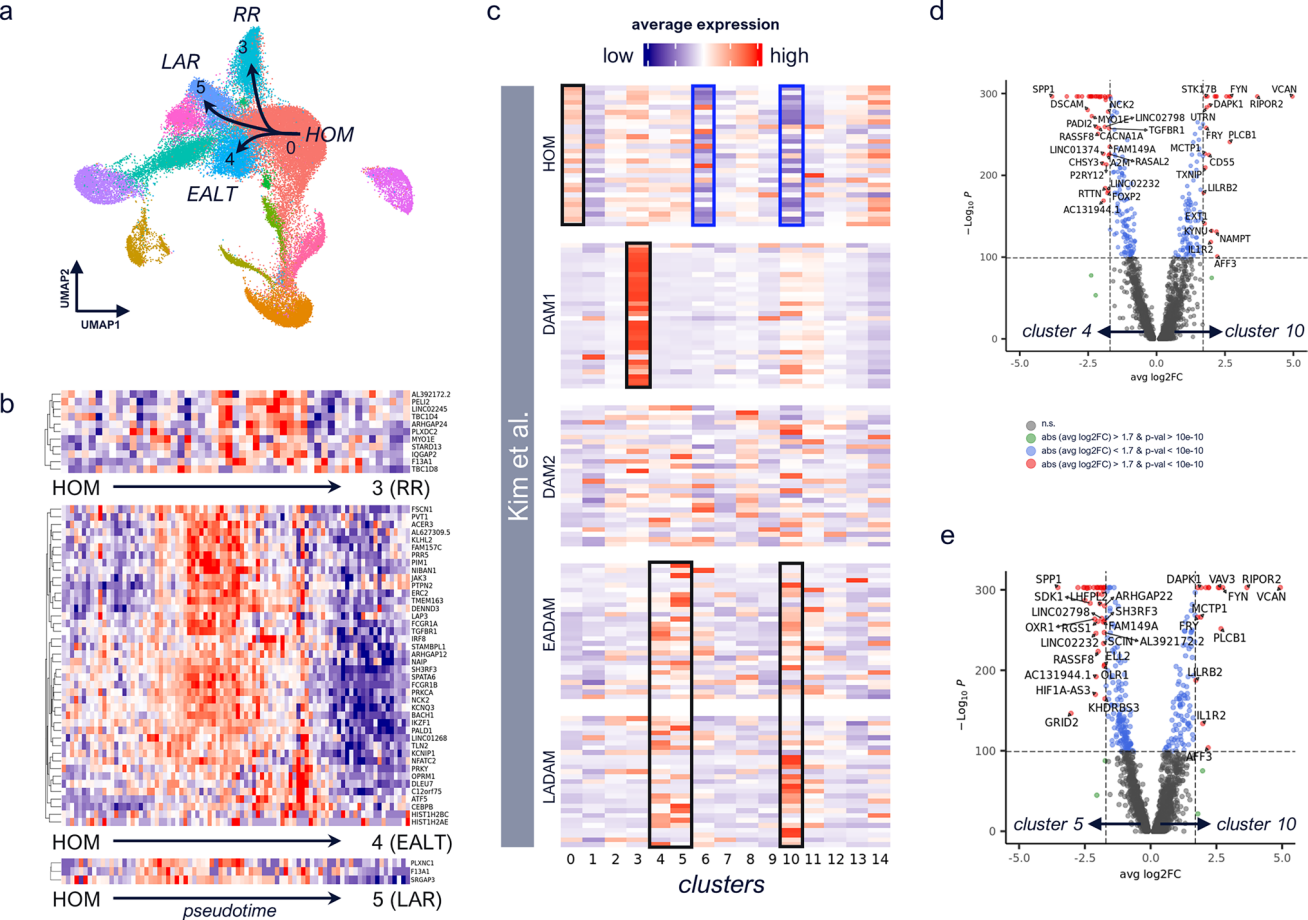
Microglia subtype conversion in human. **a** Trajectories to disease-associated clusters were identified with monocle3 [50]. **b** For individual trajectories 0 (HOM)–3 (RR), 0–4 (EALT), and 0–5 (LAR), transitionally upregulated genes were identified by splitting pseudotime into quartiles and filtering for genes expressed at a higher level in middle quartiles (transitionally higher expressed) compared to 1st and 4th one, the latter representing cluster-enriched expression in HOM, or disease-associated cluster, respectively. **c** Expression of top 30 genes per microglial phenotype (HOM, DAM1, DAM2, EADAM, LADAM) identified in [28], averaged per cluster in cross-region integrated brain myeloid cells. Red indicates high average expression levels; blue indicates low average expression levels. Clear upregulation of HOM (cluster 0) and DAM1 (cluster 3) phenotypes are observed, as well as enriched expression of LADAM and EADAM genes across clusters 4, 5, and 10, indicated by black boxes. Clusters 6 and 10 show strong relative downregulation of homeostatic microglia markers, as indicated by blue boxes. **d**,**e** Volcano plots showing genes differentially expressed between cluster 4 and 10 (**d**), and cluster 5 and 10 (**e**)

The shifts in proportion of homeostatic and AD-associated microglia clusters with increasing levels of pathology supported a transition from the former to the latter. To model human microglia transition along disease progression, we calculated trajectories and identified transitionally upregulated genes (‘phasic’ genes) in the conversion from the main homeostatic microglia cluster (cluster 0) to the AD-associated clusters identified in Fig. 3c (Fig. 4a). These suggest that human AD microglia can transition from a homeostatic (cluster 0, HOM) to either a ribosomal activation state (cluster 3, ribosomal response or RR) or AD-associated states that correlate with increases in AD pathology (clusters 4, 5) (Fig. 4b). Cluster 3 was marked by upregulation of ribosomal response-associated genes, while clusters 4 and 5 showed separate trajectories, and were designated as early Aβ/late tau (EALT), and late Aβ response (LAR), respectively, based on mapping to pathology readouts in Fig. 3c. Phasic genes from homeostatic (cluster 0) to AD-associated clusters 3, 4, and 5 included genes implicated in CDC24 GTPase cycle, RHO GTPase cycle, fibrin clot formation, IRAK1 recruitment of IKK complex (Fig. 4b, top, cluster 0 to 3), IL-4/IL-13 signaling, response of EIF2AK1 to heme deficiency, signaling by interleukins, IFNγ signaling (Fig. 4b, middle, cluster 0–4), and genes involved in axon guidance, fibrin clot formation, semaphorin interactions, and IL-4/IL-13 signaling (Fig. 4b, bottom, cluster 0 to 5). Of note, we also were able to identify trajectories from homeostatic microglia to PvM and monocyte clusters 6 and 10, respectively, indicating that microglia, PvMs and monocytes may exist on a continuum once these cells are localized within the brain parenchyma (Fig. S4a).

### Comparison with prior mouse single-cell transcriptomics studies highlights differences between microglial responses in human and mice

To determine whether microglia AD progression signatures are shared between human disease and mouse models, we compared our cross-region brain myeloid dataset to several previously reported disease-associated microglia (DAM) mouse signatures. Prior mouse scRNA-seq studies have identified a Trem2-dependent and a subsequent Trem2-independent stages of AD progression in the 5xFAD model, termed DAM1 and DAM2, respectively [27], as well as two additional DAM phenotypes, early DAM (EADAM), increased in dual Aβ and tau pathology mice, compared to single pathology mice, and late DAM (LADAM) [28]. We observed high expression of mouse homeostatic genes in our cluster 0 and of DAM1 genes in our cluster 3, and moderate expression of DAM2 genes across clusters with some expression in our clusters 5 and 8 (Fig. 4e, Fig. S4b, black boxes). Interestingly, in our data, DAM1-like cluster 3 did not precede later stage DAM2-like clusters in pseudotime (i.e., the positioning of cells along the trajectory that quantifies the relative progression of the underlying biological process), suggesting a different pathology-associated microglia transcriptional program in mouse vs. human. Remarkably, our cross-region cluster 3 contains individual region clusters with significant negative correlation with AD pathology (ITG cluster 1, PFC cluster 2), yet showed the strongest DAM1 signature (mainly ribosomal response-associated genes), suggesting that these microglia disappear with increasing pathology in human disease, which contrasts with microglial DAM1 phenotype observations in mouse models. Furthermore, expressions of EADAM and LADAM genes were not clearly delineated across human myeloid clusters, with clusters 4, 5, and 10 showing both EADAM and LADAM gene upregulation (Fig. 4c, black boxes), suggesting that this mouse early pathology-specific response may not be clearly identifiable within human donors even when analyzing multiple brain regions across a spectrum of pathology severity.

We further observed strong downregulation of mouse homeostatic microglia markers in clusters 6 and 10 (Fig. 4c, blue boxes). While cluster 6 corresponded to macrophages identified in our individual region analysis, characterized by increased expression of *LYVE1, MRC1, F13A1,* and *CD163,* cluster 10 showed increased expression of the peripheral monocyte marker *CCR2* [37]. Recent studies have identified microglia/macrophage-like cells expressing both the microglial marker *TMEM119* and the macrophage marker *CD163* surrounding Aβ plaques in human AD brains but not in control brains [40, 44]. Although microglia clusters 4 and 5 and monocyte cluster 10 were associated with EADAM and LADAM mouse microglia signatures, clusters 4 and 5 showed comparatively higher expression of canonical microglia genes *P2RY12* and *TMEM119,* and of the “AD1” gene *SPP1* (Fig. 4d, e, Table S11). Cluster 6, the PvM cluster, showed elevated *F13A1, MRC1, LYVE1,* and *CD163*, macrophage marker expression, and increased *P2RY12* as compared to monocyte cluster 10 (Fig. S4c).

In summary, some aspects of microglia transcriptomic responses to AD pathology are shared between human and mouse models, but not others.

### Pseudobulk analysis reveals genes impacted by tau, Aβ, or both, and confirms early tau dysregulation of the transcriptional regulators BACH1 and PRR5

**Fig. 5:**
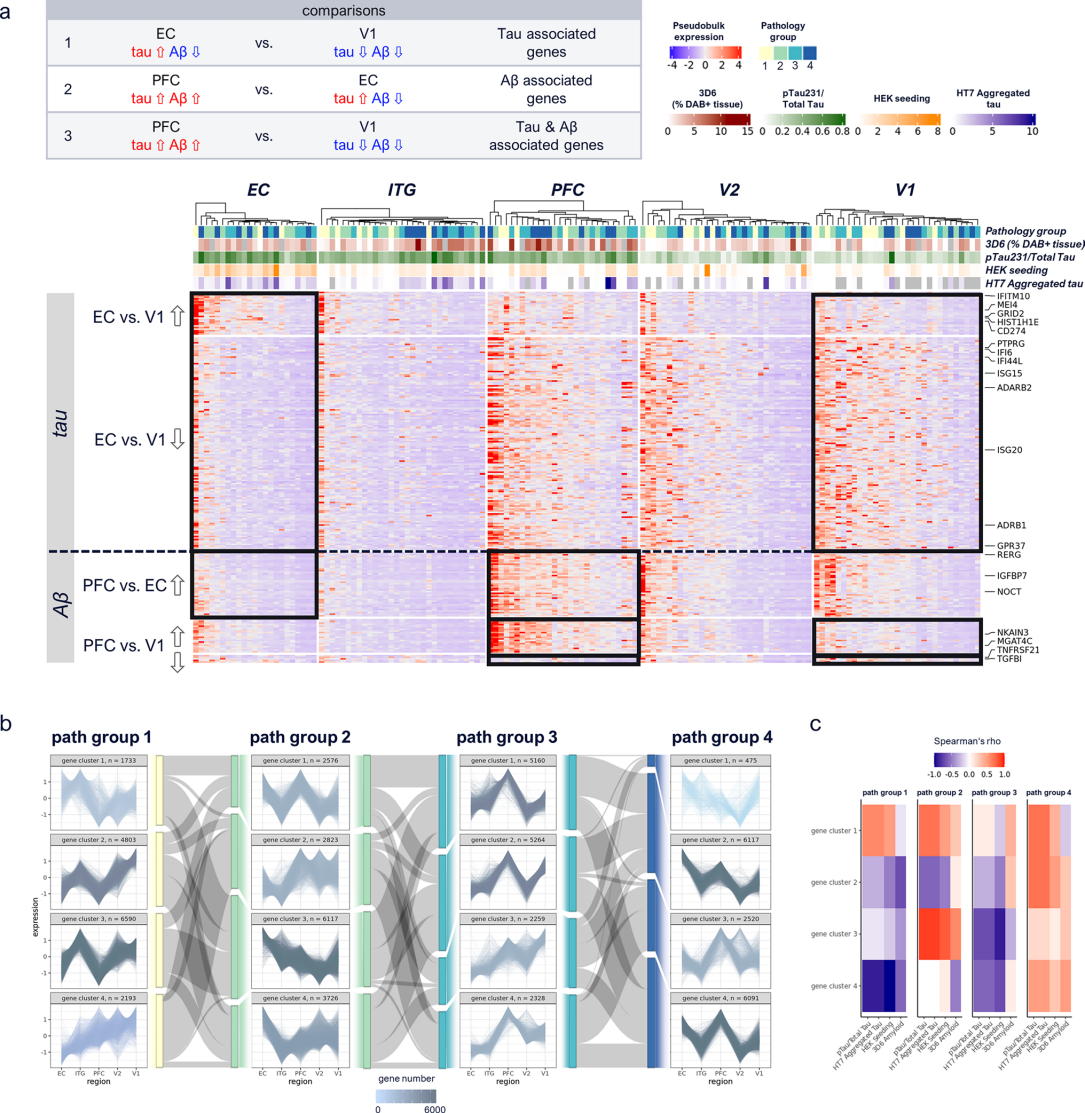
Tau- and Aβ-associated brain myeloid cell signatures. **a** Heatmap of differentially expressed genes (DEGs) up- (⇧) or down- (⇩) regulated in EC vs. V1, PFC vs. EC and PFC vs. V1 regions, within pathology group 4. Tau-driven changes (EC vs. V1) include interferon-related genes, while Aβ driven and tau & Aβ-driven changes (PFC vs. EC, PFC vs. V1) include growth factor, and cytokine signaling related genes, respectively. Results were adjusted for gender and respective pathology group 1 DEGs were filtered out. Color-coding of aggregated expression per sample (column) and gene (row), annotation shows pathology group, 3D6 Aβ IHC, pTau/Total Tau, HEK seeding, and HT7 Aggregated Tau. Filtering for DEGs based on nominal *p* value < 0.01 and logFC > 1.2. Expression patterns included in respective comparisons are indicated by black boxes; expression patterns of other regions are shown for completeness. **b** K-means gene clustering across regions, per pathology group. Gene numbers are color-coded. Sankey diagrams, color-coded according to pathology group, show percentage change of genes from given gene clusters in one pathology group to gene clusters in next pathology group. Pathology group 3 and 4 gene clusters spike in PFC region, suggesting Aβ influenced expression, while pathology group 1 and 2 contain also gene clusters showing linear correlation along regions. **c** Spearman correlation per pathology group of each gene cluster with biochemical readouts; overall highest correlation is observed in pathology group 4, across gene clusters. High correlation is indicated by red and low correlation by blue color

While analysis at the single-cell level is a powerful tool to characterize microglial phenotypes based on cell-to-cell variation, single cells from the same tissue sample cannot be considered truly independent sample. Leveraging the cohort size, we were interested in expanding our analysis to also identify disease-associated genes at a population level. To confirm tau vs. tau & Aβ driven changes in late-stage AD brain myeloid cells, we compared pseudobulk gene expression in high-tau/low Aβ vs. low-tau/low-Aβ (EC vs. V1, tau-driven), high-tau/high-Aβ vs. high-tau/low-Aβ (PFC vs. EC, Aβ-driven), and high-tau/high-Aβ vs. low-tau/low-Aβ (PFC vs. V1, tau & Aβ-driven) regions within high pathology group 4 donors, controlling for regional changes observed in low-pathology group 1 donors (Fig. 5a, Table S12). Tau-driven changes included interferon-related genes (*IFITM10, IFI44L, and IFG20*), which were also increased in tau-associated clusters from our single-cell level analysis (e.g*., IFI44L* in cross-region tau-associated cluster 2 vs. cluster 0), as well as the previously “AD2” identified gene *GRID2*. Tau & Aβ-driven changes included genes related to cytokine (*TNFRSF21, TGFBI*) signaling. Clustering of genes across regions per pathology group demonstrated that the majority of pathology group 3 and 4 genes spike in PFC, suggesting mainly Aβ-influenced signatures later in disease progression. These included pathology group 3 gene cluster 2, represented by pathways such as “antigen processing: ubiquitination & proteasome degradation” (Fig. 5b, Table S13, S5a, Table S14). On the other hand, pathology groups 1 and 2 had gene clusters following tau progression (high EC–low V1 expression or low EC–high V1 expression), e.g., pathology group 1 gene cluster 4, mainly corresponding to pathology group 2 gene cluster 2 and represented by pathways such as “extracellular matrix organization” (Fig. 5b, Fig. S5a). Correlation of each gene cluster with biochemical readouts indicated overall highest correlation in pathology group 4, across gene clusters (Fig. 5c). Additionally, pathology group 2, gene cluster 3 showed positive correlations with all pathology readouts, while pathology group 3 gene cluster 2 showed positive correlations with Aβ but not tau, as expected based on gene expression patterns of EC > ITG > PFC > V2/V1 and EC < ITG < PFC > V2/V1, respectively. We further sought to identify genes that showed opposite expression patterns in early disease stages (path group 1 compared to path group 2), indicating early tau-associated dysregulation, which included the transcriptional regulators *BACH1* and *PRR5* (Fig. S5b, Table S15). Notably, genes previously identified as transitionally upregulated in the conversion from cluster 0 (HOM) to 4 (EALT) showed significant overlap (p value 1.74e-5) with genes showing early tau pathology-driven dysregulation, e.g., *BACH1* and *PRR5*, thus supporting the validity of our trajectory results.

## Discussion

This study analyzes the highest number of microglia and brain macrophages in human AD at the single-cell level to date. It leverages additional biochemical and immunohistochemical readouts from the same tissue samples, making it a unique and comprehensive data resource to understand the AD landscape of brain myeloid cells across tau progression [1, 14, 30, 41]. In a cohort of 32 human donors, we characterized the progression of AD pathology, both temporally, from low to high pathology, and spatially, from more to less vulnerable regions.

While here, we focused on brain myeloid cells, our parent study included snRNA-seq of all brain cell types. We found that microglia are relatively uniform across the neocortex, whereas astrocytes and endothelial cells have brain region-specific signatures [6, 46]. Astrocytes develop an apparent decreased activation (“burnt out”) phenotype in end-stage disease [46], whereas we see no evidence of an analogous state for microglia.

Entorhinal cortex, part of the periallocortex, is one of the first regions typically affected by tau pathology in AD and displays a different cortical layer structure compared to neocortex. We found that while most brain myeloid cells are similar between neocortical regions (ITG, PFC, V1, V2), there was a subpopulation of EC microglia showing a distinct signature, with genes involved in vesicle and potassium ion transport. Whether or not these differences relate to functionally distinct allocortical microglia is unclear, but these data suggest that there are region-specific phenotypes of microglia in the human brain that we observed are neither specific to donors with high or low pathology, nor enriched for Aβ or tau pathology readouts.

Across the five brain regions, we identified clusters of microglia that were both positively or negatively correlated with tau and/or Aβ pathology. From this dataset, it is impossible to determine whether positively correlated clusters are cause or consequence of pathology, since human postmortem brain snRNA-seq studies are inherently cross-sectional, and AD progression likely involves intricate interactions between all brain cell types including astrocytes, neurons, and those of the vasculature [10]. On the other hand, microglia clusters negatively correlated with pathology suggest either microglial degeneration or transition to alternate phenotypes. Our analysis points to the latter as it indicates transition of homeostatic microglia to multiple disease-associated states, and we found no transcriptional evidence of microglial apoptosis or senescence. However, we filtered out nuclei with high mitochondrial content, which in cells indicates cell death, so our dataset may not fully characterize degenerating cells.

Besides sampling different brain regions, our study included multiple biochemical assays for pathological tau, such as phosphorylated tau, aggregated tau, and propensity for tau seeding, which increased our ability to identify tau-associated microglia at different stages of pathological tau progression. Building on previous studies, e.g., [15], our study encompasses not only microglia but also brain macrophages, including perivascular macrophages. Perivascular macrophages have been suggested to trigger neurovascular dysfunction in AD through release of reactive oxygen species [43]. Although PvMs are normally localized to the Virchow–Robin space around blood vessels and differ from microglia by expression of markers including *CD163*, recent studies have identified microglia/macrophage-like cells expressing both the microglial marker *TMEM119* and the macrophage marker *CD163* surrounding Aβ plaques in human AD brain but not in control brains [40, 44]. It has been suggested that peripheral monocytes become PvMs when the blood–brain barrier is disrupted [37] and that blood–brain barrier damage starts early in the course of AD [24, 38]. This suggests that macrophages, including PvMs, migrate toward plaques or that microglia differentiate into macrophage-like cells during AD progression and accumulate to a significant degree in donors with high AD pathology. Of note, we calculated trajectories from homeostatic microglia to the macrophage-like clusters 6 and 10 in our cross-region dataset, suggesting that microglia could exist on a continuum with brain macrophages; however, this in silico observation needs to be experimentally validated. In contrast to our study, Gerrits et al. removed brain macrophages from their analysis, which may explain some of the differences between the studies. Future human brain snRNA-seq studies should carefully consider how microglia and brain macrophages are analyzed and differentiated, as both cell types likely contribute to the AD disease course and can be therapeutic targets.

The three distinct microglia trajectory endpoints were marked by upregulation of genes including *SPP1* (cluster 4)*. SPP1,* encoding the secreted phosphoprotein osteopontin, has been implicated in microglia-mediated synaptic engulfment [45]. Functional studies involving genes in microglia trajectories and endpoints will help determine microglial involvement, either directly via tau processing, or indirectly through secretion of soluble factors, in tau progression and associated sequelae including synaptic loss.

In contrast with mouse datasets [27, 28], we found that human AD microglia can transition from homeostatic to a state associated with low AD pathology and characterized by upregulation of ribosomal genes (ribosomal response or RR), or to other states associated with increased AD pathology (early Aβ, late tau or EALT, and late Aβ response or LAR). Of note, part of the human AD microglial response is clearly modeled by the single Aβ pathology model (“DAM1”, derived from the 5xFAD model, corresponding to our cross-region cluster 3). Three of our additional human microglia clusters also correlate with EADAM and LADAM signatures from the dual pathology Aβ & tau mouse model. However, these differences suggest a need for better animal modeling of the unique spatial and temporal interaction of human tau progression with Aβ deposition. On a technical level, some of the mouse vs. human differences could be due to differential detection of transcripts in cells vs. nuclei, as the mouse datasets used scRNA-seq, while the human datasets used snRNA-seq. Finally, our study focused on transcriptional changes in myeloid cell subtypes. We investigated several cluster markers at the protein level (TMEM119, CPM, and CD163) and found similar patterns of gene and protein expression for these markers. However, further study would be required to determine global correlations in gene and protein expression in brain myeloid subtypes at the single-cell level, which is beyond current available proteomics technology.

Finally, we found that in early stages, (e.g., pathology groups 2 vs. 1), tau pathology is the main driver of microglial transcriptional signatures, while in later stages (e.g., pathology groups 3 and 4), microglial transcriptional changes are also strongly associated with Aβ plaque burden. We used early progression expression patterns to identify genes that reversed their pattern early in the stereotypical spatial progression of pTau neurofibrillary tangles (EC > ITG > PFC > V2 > V1) and identified their potential involvement in microglia subtype conversion to a diseased state (cluster 4, *EALT*). Among them, the transcriptional regulators *BACH1* and *PRR5* stood out. BACH1 has been proposed as a therapeutic target for several neurological diseases (e.g., Parkinson’s disease, multiple sclerosis), and PRR5 is part of the mTORC2 protein complex that is decreased in the AD brain at the protein level [1, 29, 36, 54]. Thus, targeting these genes, or their upstream/downstream pathways, could slow gene programs initiated by early progression of tau pathology.

Although this dataset represents the largest high-quality and high-information published human microglia single-cell study to date, the major challenge remains: Human tissue cannot be sampled in a longitudinal fashion, and postmortem autopsies necessitate use of age-matched disease-free controls. Consequently, we could not include tau-negative controls in our study, as aged human brains rarely have no tau pathology in the EC [41, 49]. We took two approaches to partially overcome these challenges: (1) We split the cohort into four pathology groups, which allowed us to cross-compare donors with less tau pathology vs. donors with more tau pathology, and (2) We harnessed the power of snRNA-seq and used trajectory analysis to model in silico disease progression [52].

In summary, we identified previously unknown regional microglia states, as well as early and late disease-associated microglia signatures across brain regions, and uncovered human microglia transitions associated with pathology progression in AD. From a homeostatic state human microglia either develop into a ribosomal response state, similar to that of mouse DAM1, or into distinct pathology-associated states. However, unlike in mouse, the ribosomal response state does not precede later stage disease states but is of a different trajectory. We propose these microglia states as the focus of future functional studies to determine whether interference can halt, or at least stall, the progression of human AD.

### Supplementary Information

Below is the link to the electronic supplementary material.

Fig. S1 (related to Figure 1). a) Study cohort information. b) Example FACS plot showing gating and enrichment strategy. c) Total tau quantification per pathology groups across regions. d) Representative CD11c (ITGAX) IHC (EC, grey matter), with CD11c in brown and Aβ plaques (3D6) in red (scale bar 100 µm). e) Representative CD68 IHC (EC, grey matter), with CD68 in brown and plaques (D54D2) in red (scale bar 100 µm). Fig. S2 (related to Figure 2). a) Distribution of donor IDs per cluster. Different donor numbers per brain region indicate if tissue was not available from all brain regions for each donor. Clusters with individual donor contribution > 75% were considered donor-specific (indicated by black boxes) and disregarded in downstream analyses and interpretation. b) Overlap of up- and down-regulated brain myeloid cell markers per region (vs. other regions). The number of differentially expressed genes is <0.3% of all annotated genes, across regions. EC shows the highest number of both differentially up- and downregulated genes compared to all other regions. c) Spearman correlation of aggregated expression per brain region x cluster (excluding donor-specific clusters) shows similar expression levels between regions (‘*’ indicates p-value < 0.001); overlap of detected genes (>0 UMI counts in >0.1% of microglia nuclei per region) shows high similarity of gene detection, with slightly higher number of uniquely detected genes in PFC and lower number in ITG compared to other brain regions. d) EC enriched population in Fig. 1F was compared to other EC region cells. Similar biological process GO terms were found enriched compared to a cross-region brain myeloid cell comparison. e) Per region proportion of individual donors in EC enriched subcluster 4. f) Per region number of cluster 4 nuclei, color-coded by pathology group (left), and per region proportion of pathology groups within cluster 4 (right). Fig. S3 (related to Figure 3). a) Per region Spearman correlation of brain myeloid cell clusters (differentially expressed genes per cluster vs. homeostatic cluster 0 per region) with public genelists. Significant correlation indicated by *** p-value < 0.001, **p-value < 0.01, *p-value < 0.05, grey boxes indicate insufficient data (number of overlapping genes between data sets < 10). AD1 and AD2 signatures from [16]; CRM2 (cytokine response 2), CYT/CRM1 (cytokine response 1), DAM (disease associated), HLA (antigen-presenting response), HM (homeostatic), IRM (Interferon response), RM (ribosomal response), TRANS (transitioning CRM) signatures from [33]; and tau fibril response genes from [52]. For reference, solid black boxes denote clusters positively correlated with pathology in Fig 2A; dashed black boxes denote clusters negatively correlated with pathology in Fig 2A. b-d) Representative amyloid plaques (3D6) and tau tangles (AT100) along with microglia markers TMEM119 (b) and CPM (c), and PvM marker CD163 (d) IHC (ITG, grey matter) of pathology group 3 or 4 and pathology group 1 samples (scale bars 100 µm). Arrows and arrowhead in d) indicate CD163-positive cell with monocyte/macrophage-like morphology in brain vasculature, and CD163-positive cell in the brain parenchyma, respectively. e) Quantification of IHC data shown in b-d, from 1 brain section per donor across 4 pathology groups. Plot shows median and first and third quartiles. f) Per region mapping of region-specific clusters (upper panel UMAP plots) to cross-region integrated data object (lower panel UMAP plots). Color-coding based on clusters identified per region. Solid black circles denote clusters positively correlated with pathology in Fig. 2a; dashed black circles denote clusters negatively correlated with pathology in Fig. 2a, for reference. ”NA“ signifies brain myeloid cells in the cross-region dataset from regions other than the respective comparison region shown in region-specific UMAP plots. g) Differential gene expression of cross-region integrated clusters vs. HOM cluster 0, indicated by text are top3 up- and downregulated differentially expressed genes per cluster. Black boxes highlight clusters identified as disease-associated in Fig. 2a. h) Feature plots of top 3 markers differentiating clusters more associated with tau (clusters 2 and 4) vs. clusters more associated with Aβ (clusters 5 and 8). i) Reactome pathway enrichment results for cross-region integrated brain myeloid cells per cluster (cluster vs. HOM cluster 0), filtered to top3 enriched pathways per cluster and >= 5 genes per pathway. Black boxes highlight clusters identified as disease-associated, cluster 4 did not show pathway enrichment based on filtering criteria. j) Feature plots showing perivascular macrophage markers LYVE1, MRC1, CD163 and peripheral myeloid cell marker CCR2. Fig. S4 (related to Figure 4). a) Trajectories to disease associated clusters were identified with monocle3 (Trapnell et al., 2014), including traejectories to perivascular macrophage cluster 6 (PvM) and monocyte cluster 10 (Mono). b) Expression of microglial phenotype markers (HOM, DAM1, DAM2) as described [27], averaged per cluster in cross-region integrated microglia. Red indicates high average expression levels, blue indicates low average expression levels. No strong association with clusters from this study, but moderate expression of DAM1 genes in cluster 3, and DAM2 genes in clusters 5 and 8, as indicated by black boxes c) Volcano plot showing genes differentially expressed between PvM cluster 6 and monocyte cluster 10. Fig. S5 (related to Figure 5). a) Top 5 Reactome pathways per gene cluster and pathology group, as shown in Fig. 3b (adj. p-values < 0.05). b) Selected genes (BACH1, PRR5) showing opposite expression patterns in early disease stages (path group 1 compared to path group 2), indicating early tau pathology driven dysregulation, and upregulation in PFC in later disease stages (path groups 3 and 4), indicating late primarily Aβ driven dysregulation. Supplementary file1 (PDF 2572 kb)

Table S1. Donor metadata. Table S2. Differential gene expression per region in cross-region integrated data. Table S3. Gene detection per region. Table S4. Differential gene expression per brain region, per cluster (vs. other clusters). Table S5. Reactome pathway enrichment per brain region, per cluster (vs. HOM cluster). Table S6. Differential gene expression per cluster vs. homeostatic microglia, in cross-region integrated data. Table S7. Differential gene expression between clusters 2+4 vs. clusters 5+8, in cross-region integrated data. Table S8. Reactome pathway enrichment per cluster vs. homeostatic microglia, in cross-region integrated data. Table S9. Sequential pairwise differential gene expression between pathology readout bins, in cross-region integrated data. Table S10. Reactome pathway enrichment for sequential comparisons between pathology readout bins, in cross-region integrated data. Table S11. Differential gene expression between clusters 4, 5 and 6 vs. cluster 10, in cross-region integrated data. Table S12. Pseudobulk region comparison results in pathology group 4 donors (in cross-region integrated data), pathology group 1 donor differentially expressed genes were removed. Table S13. Gene clusters per pathology group. Table S14. Reactome pathway enrichment of gene clusters per pathology group. Table S15. Gene clusters showing reversal of expression program in early disease stages (pathology group 1 vs pathology group 2) across brain regions; respective Reactome enrichment analysis results. Supplementary file2 (XLSX 128264 kb)

## Data Availability

Raw data of this study are available via SRA accession PRJNA916657 (https://www.ncbi.nlm.nih.gov/bioproject/PRJNA916657/). Processed data can be browsed at https://ad-progression-atlas.partners.org.
