# Supplementary material for: Landscape of brain myeloid cell transcriptome along the spatiotemporal progression of Alzheimer’s disease reveals distinct sequential responses to Aβ and tau
Source: Acta Neuropathol. 2024 Apr 1;147(1):65. doi: 10.1007/s00401-024-02704-2 (PMC10984903; doi:10.1007/s00401-024-02704-2)
Supplement: Supplementary file 1 — Fig. S1 (related to Figure 1). a) Study cohort information. b) Example FACS plot showing gating and enrichment strategy. c) Total tau quantification per pathology groups across regions. d) Representative CD11c (ITGAX) IHC (EC, grey matter), with CD11c in brown and Aβ plaques (3D6) in red (scale bar 100 µm). e) Representative CD68 IHC (EC, grey matter), with CD68 in brown and plaques (D54D2) in red (scale bar 100 µm). Fig. S2 (related to Figure 2). a) Distribution of donor IDs per cluster. Different donor numbers per brain region indicate if tissue was not available from all brain regions for each donor. Clusters with individual donor contribution > 75% were considered donor-specific (indicated by black boxes) and disregarded in downstream analyses and interpretation. b) Overlap of up- and down-regulated brain myeloid cell markers per region (vs. other regions). The number of differentially expressed genes is <0.3% of all annotated genes, across regions. EC shows the highest number of both differentially up- and downregulated genes compared to all other regions. c) Spearman correlation of aggregated expression per brain region x cluster (excluding donor-specific clusters) shows similar expression levels between regions (‘*’ indicates p-value < 0.001); overlap of detected genes (>0 UMI counts in >0.1% of microglia nuclei per region) shows high similarity of gene detection, with slightly higher number of uniquely detected genes in PFC and lower number in ITG compared to other brain regions. d) EC enriched population in Fig. 1F was compared to other EC region cells. Similar biological process GO terms were found enriched compared to a cross-region brain myeloid cell comparison. e) Per region proportion of individual donors in EC enriched subcluster 4. f) Per region number of cluster 4 nuclei, color-coded by pathology group (left), and per region proportion of pathology groups within cluster 4 (right). Fig. S3 (related to Figure 3). a) Per region Spearman correlation of [file 401_2024_2704_MOESM1_ESM.pdf]

Figure S1

a

	Pathology Group 1	Pathology Group 2	Pathology Group 3	Pathology Group 4
Definition	Braak 0-II<br>Thal 0	Braak II-III<br>Thal 1-4	Braak V<br>Thal 3-5	Braak VI<br>Thal 3-5
Variable				
**Sex: n (%)**				
**Female**	4 (50)	5 (62.5)	6 (75)	4 (50)
**Male**	4 (50)	3 (37.4)	2 (25)	4 (50)
**Age (yrs)*: mean (SD)**	79.5 (12.3)	86.0 (6.6)	82.9 (7.9)	79.5 (10.4)
**PMI (hrs): mean (SD)**	18.4 (6.8)	22.1 (11.3)	18.3 (11.5)	15.6 (7.1)
**APOE status: n (%)**				
**2/2**	0 (0)	1 (12.5)	0 (0)	0 (0)
**2/3**	0 (0)	1 (12.5)	1 (12.5)	0 (0)
**3/3**	8 (100)	6 (75)	4 (50)	4 (50)
**3/4**	0 (0)	0 (0)	2 (25)	4 (50)
**4/4**	0 (0)	0 (0)	1 (12.5)	0 (0)
**Brain regions: n (%)**				
**EC**	3 (37.5)	7 (87.5)	6 (75)	7 (87.5)
**ITG**	7 (87.5)	8 (100)	8 (100)	8 (100)
**PFC**	4 (50)	8 (100)	8 (100)	8 (100)
**V2**	8 (100)	8 (100)	8 (100)	8 (100)
**V1**	8 (100)	8 (100)	8 (100)	8 (100)

* Donors of age 90+ yrs were considered being of age 90 yrs for calculation of mean/SD only.

b

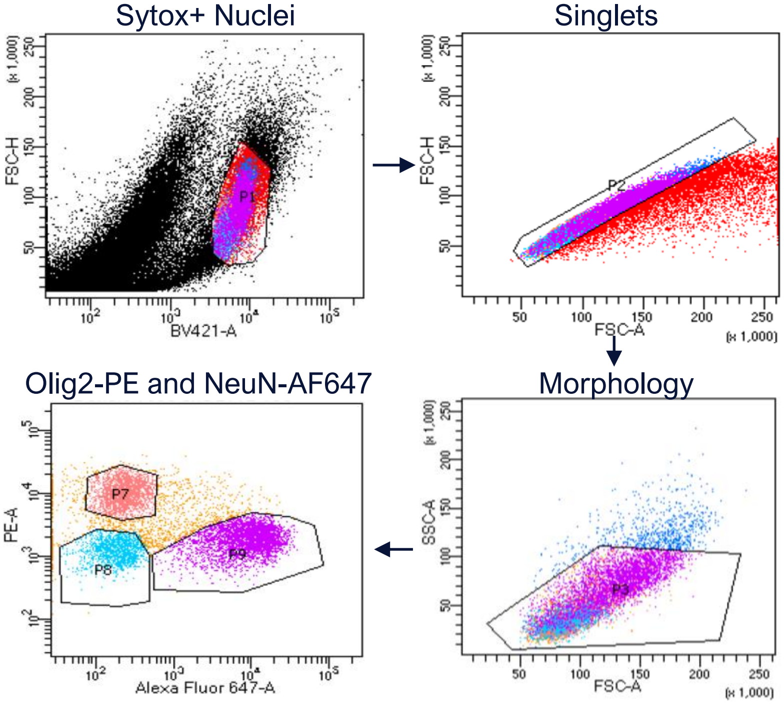

c

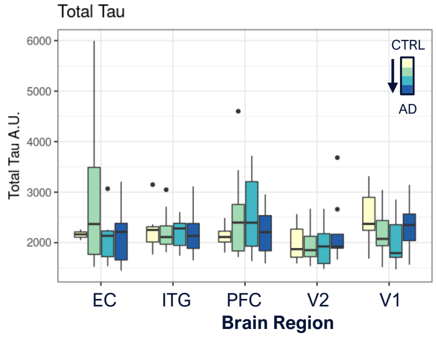

d

CD11c

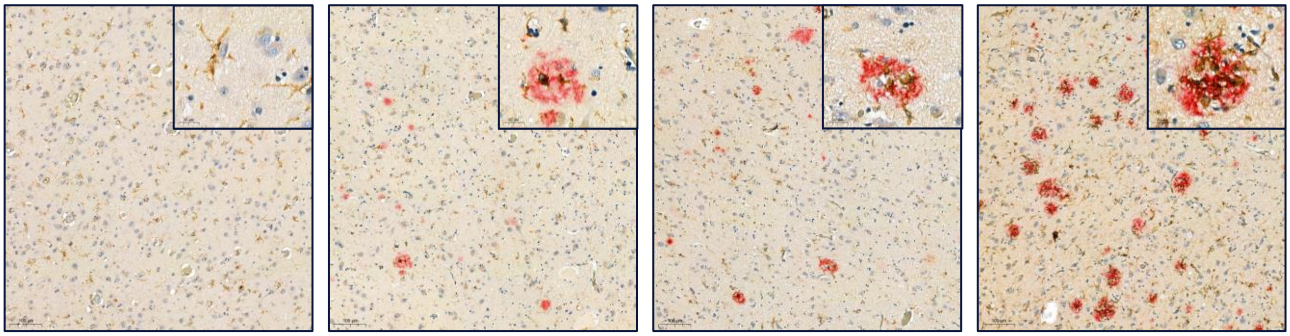

Pathology group 1 Pathology group 2 Pathology group 3 Pathology group 4

e

CD68

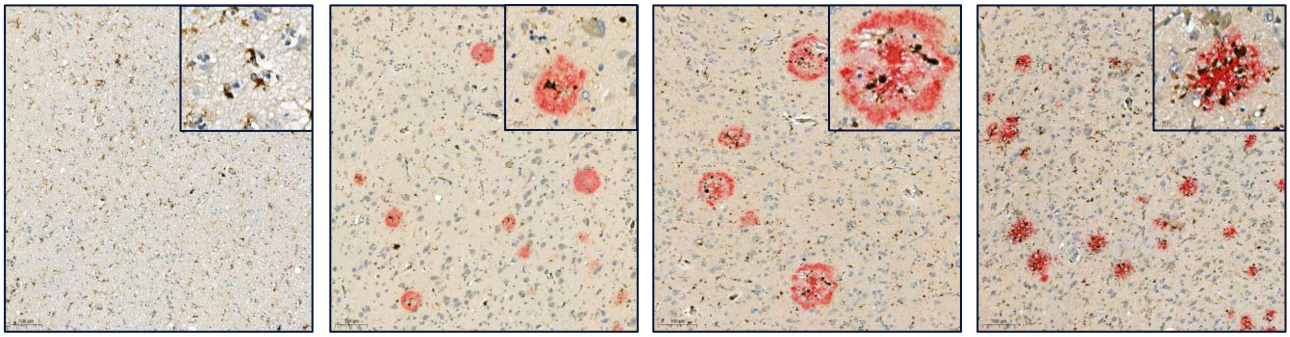

Pathology group 1 Pathology group 2 Pathology group 3 Pathology group 4

Figure S2

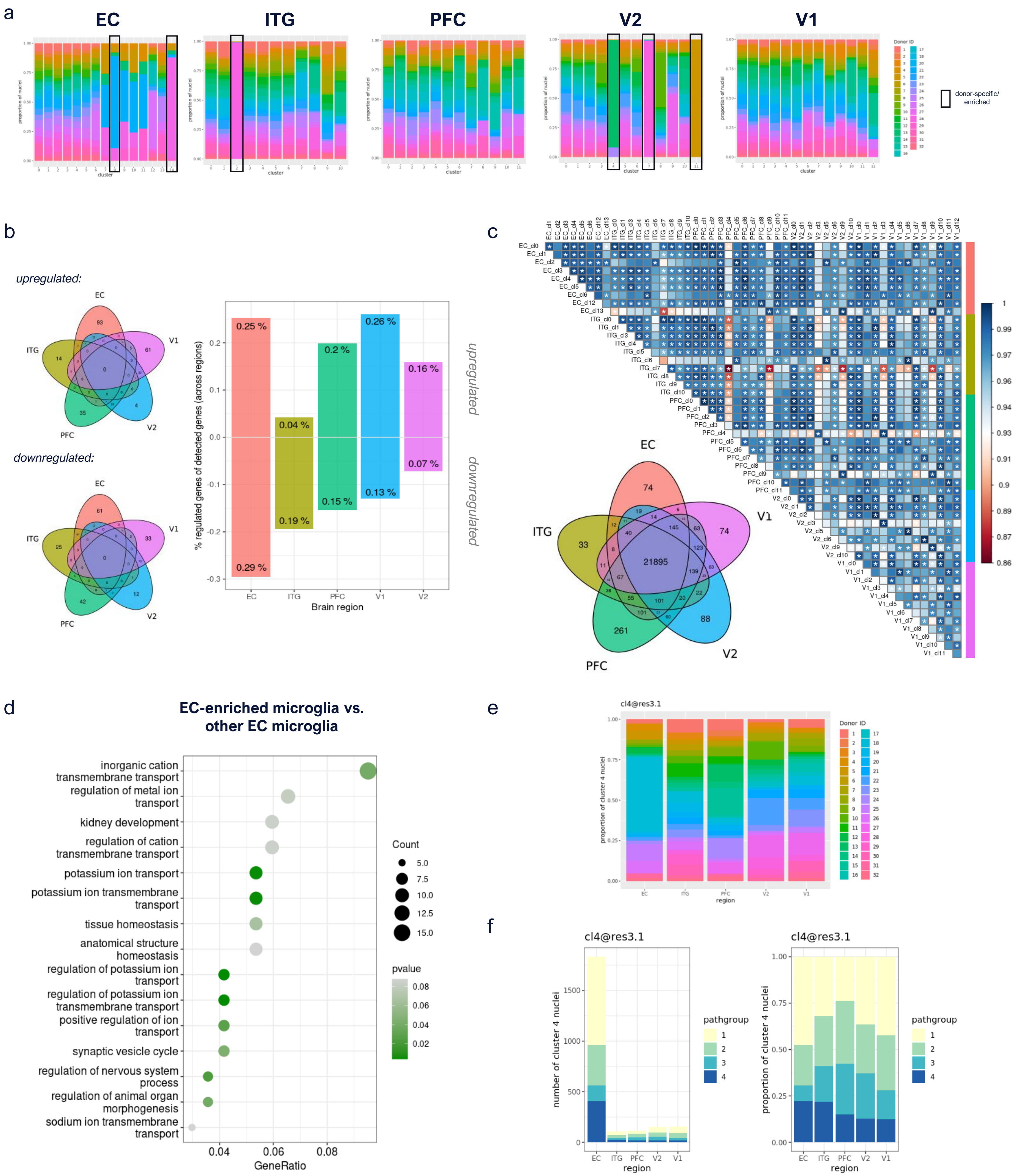

a
EC
ITG
PFC
V2
V1
Donor ID
donor-specific/ enriched
b
upregulated:
downregulated:
% regulated genes of detected genes (across regions)
Brain region
upregulated
downregulated
c
d
EC-enriched microglia vs. other EC microglia
inorganic cation transmembrane transport
regulation of metal ion transport
kidney development
regulation of cation transmembrane transport
potassium ion transport
potassium ion transmembrane transport
tissue homeostasis
anatomical structure homeostasis
regulation of potassium ion transport
regulation of potassium ion transmembrane transport
positive regulation of ion transport
synaptic vesicle cycle
regulation of nervous system process
regulation of animal organ morphogenesis
sodium ion transmembrane transport
GeneRatio
Count
pvalue
e
cl4@res3.1
proportion of cluster 4 nuclei
region
f
cl4@res3.1
number of cluster 4 nuclei
pathgroup
region

Figure S3

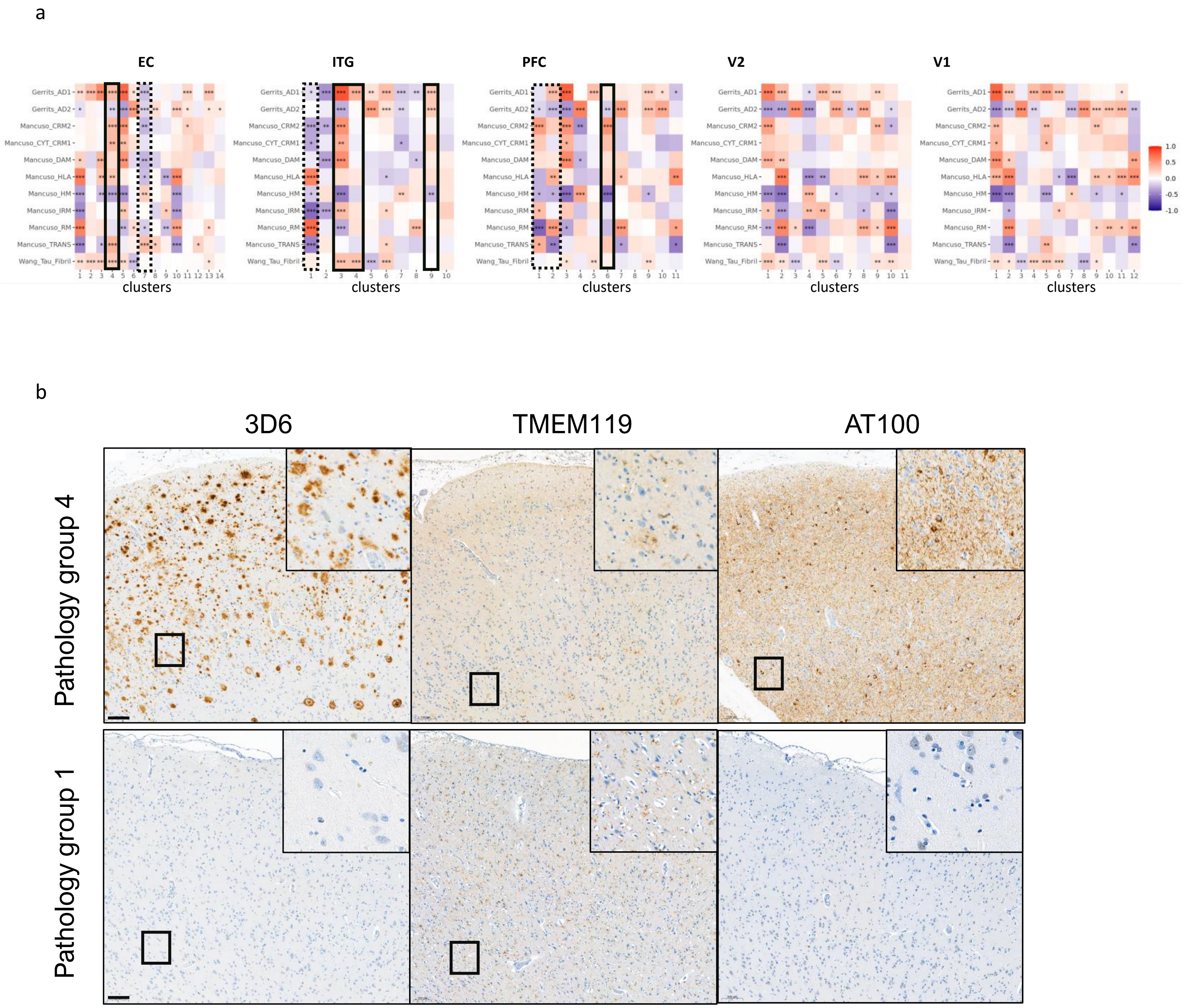
a
EC
ITG
PFC
V2
V1
Gerrits_AD1
Gerrits_AD2
Mancuso_CRM2
Mancuso_CYT_CRM1
Mancuso_DAM
Mancuso_HLA
Mancuso_HM
Mancuso_IRM
Mancuso_RM
Mancuso_TRANS
Wang_Tau_Fibril
clusters
1.0
0.5
0.0
-0.5
-1.0
b
3D6
TMEM119
AT100
Pathology group 4
Pathology group 1

Figure S3

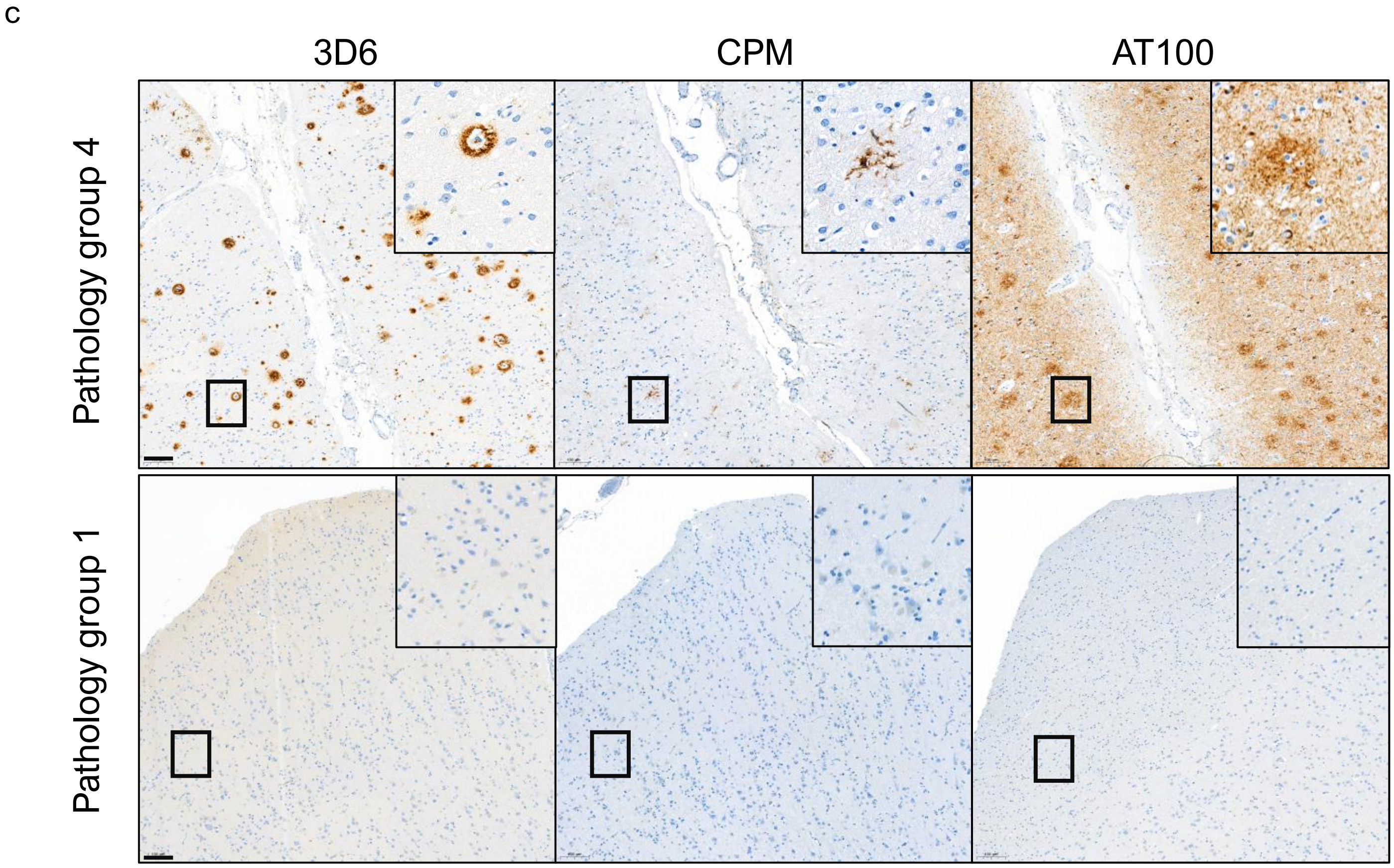
c
3D6
CPM
AT100
Pathology group 4
Pathology group 1

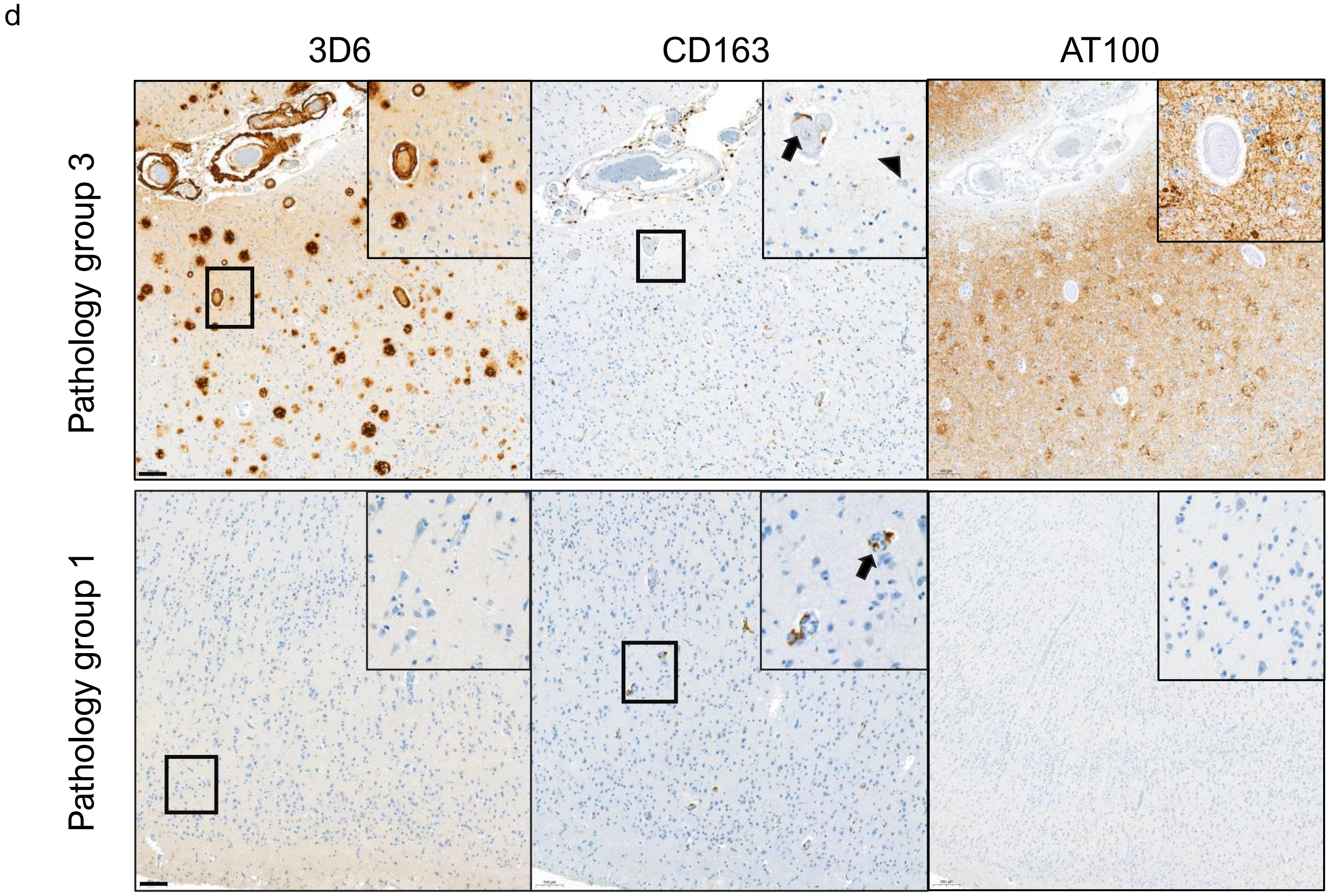
d
3D6
CD163
AT100
Pathology group 3
Pathology group 1

Figure S3

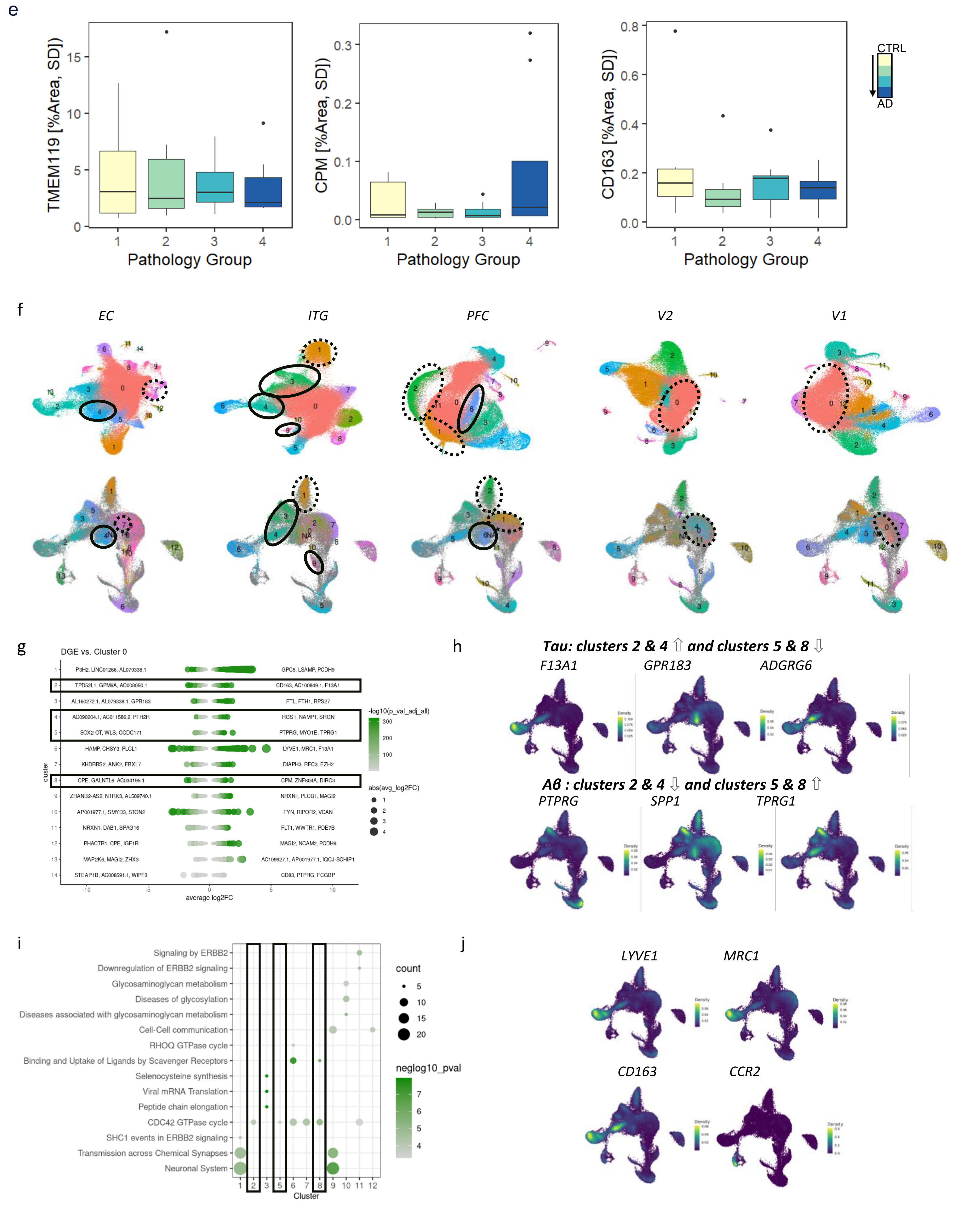

e
TMEM119 [%Area, SD])
CPM [%Area, SD])
CD163 [%Area, SD])
Pathology Group
CTRL
AD
f
EC
ITG
PFC
V2
V1
g
DGE vs. Cluster 0
P3H2, LINC01266, AL079338.1
GPC5, LSAMP, PCDH9
TPD52L1, GPM6A, AC008050.1
CD163, AC100849.1, F13A1
AL160272.1, AL079338.1, GPR183
FTL, FTH1, RPS27
AC090204.1, AC011586.2, PTH2R
RGS1, NAMPT, SRGN
SOX2-OT, WLS, CCDC171
PTPRG, MYO1E, TPRG1
HAMP, CHSY3, PLCL1
LYVE1, MRC1, F13A1
KHDRBS2, ANK2, FBXL7
DIAPH3, RFC3, EZH2
CPE, GALNTL6, AC034195.1
CPM, ZNF804A, DIRC3
ZRANB2-AS2, NTRK3, AL589740.1
NRXN1, PLCB1, MAGI2
AP001977.1, SMYD3, STON2
FYN, RIPOR2, VCAN
NRXN1, DAB1, SPAG16
FLT1, WWTR1, PDE7B
PHACTR1, CPE, IGF1R
MAGI2, NCAM2, PCDH9
MAP2K6, MAGI2, ZHX3
AC109927.1, AP001977.1, IQCJ-SCHIP1
STEAP1B, AC008591.1, WIPF3
CD83, PTPRG, FCGBP
cluster
average log2FC
-log10(p_val_adj_all)
abs(avg_log2FC)
h
Tau: clusters 2 & 4 ⇧ and clusters 5 & 8 ⇩
F13A1
GPR183
ADGRG6
Aβ : clusters 2 & 4 ⇩ and clusters 5 & 8 ⇧
PTPRG
SPP1
TPRG1
Density
i
Signaling by ERBB2
Downregulation of ERBB2 signaling
Glycosaminoglycan metabolism
Diseases of glycosylation
Diseases associated with glycosaminoglycan metabolism
Cell-Cell communication
RHOQ GTPase cycle
Binding and Uptake of Ligands by Scavenger Receptors
Selenocysteine synthesis
Viral mRNA Translation
Peptide chain elongation
CDC42 GTPase cycle
SHC1 events in ERBB2 signaling
Transmission across Chemical Synapses
Neuronal System
Cluster
count
neglog10_pval
j
LYVE1
MRC1
CD163
CCR2

Figure S4

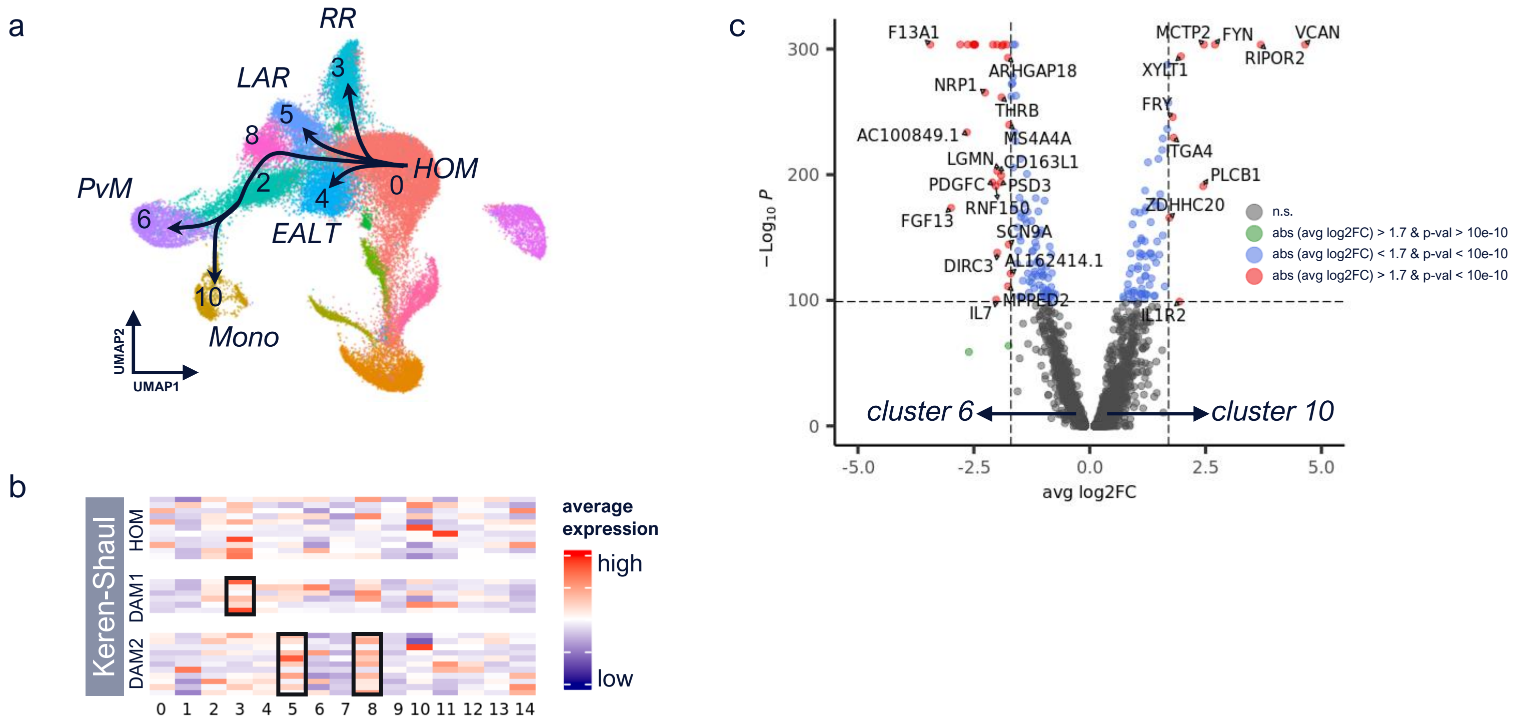
a
RR
LAR
PvM
HOM
EALT
Mono
3
5
8
2
0
4
6
10
UMAP2
UMAP1
b
Keren-Shaul
HOM
DAM1
DAM2
0 1 2 3 4 5 6 7 8 9 10 11 12 13 14
average expression
high
low
c
F13A1
ARHGAP18
NRP1
THRB
AC100849.1
MS4A4A
LGMN
CD163L1
PDGFC
PSD3
RNF150
FGF13
SCN9A
DIRC3
AL162414.1
MPPED2
IL7
MCTP2
FYN
VCAN
RIPOR2
XYLT1
FRY
ITGA4
PLCB1
ZDHHC20
IL1R2
n.s.
abs (avg log2FC) > 1.7 & p-val > 10e-10
abs (avg log2FC) < 1.7 & p-val < 10e-10
abs (avg log2FC) > 1.7 & p-val < 10e-10
cluster 6
cluster 10
avg log2FC
-5.0
-2.5
0.0
2.5
5.0
300
200
100
0

Figure S5

a

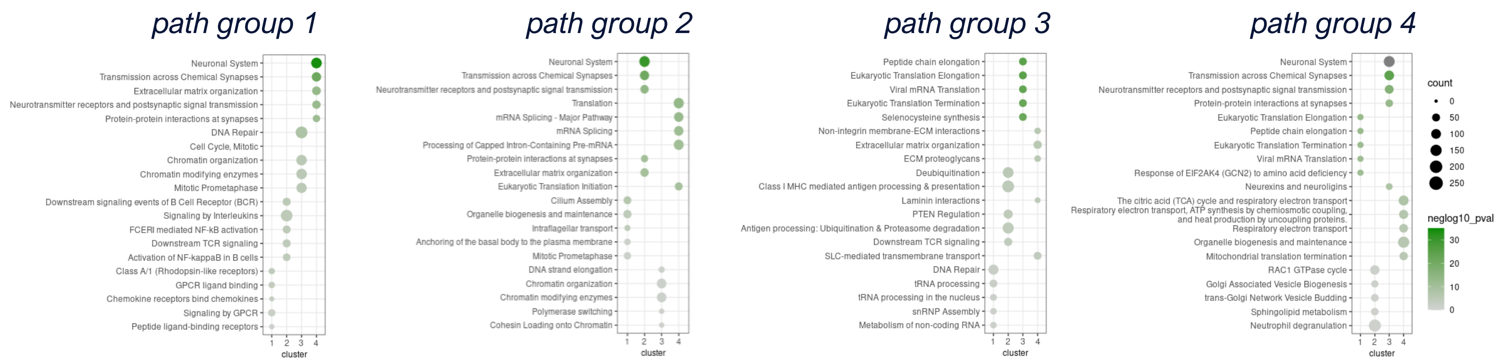

path group 1
Neuronal System
Transmission across Chemical Synapses
Extracellular matrix organization
Neurotransmitter receptors and postsynaptic signal transmission
Protein-protein interactions at synapses
DNA Repair
Cell Cycle, Mitotic
Chromatin organization
Chromatin modifying enzymes
Mitotic Prometaphase
Downstream signaling events of B Cell Receptor (BCR)
Signaling by Interleukins
FCERI mediated NF-kB activation
Downstream TCR signaling
Activation of NF-kappaB in B cells
Class A/1 (Rhodopsin-like receptors)
GPCR ligand binding
Chemokine receptors bind chemokines
Signaling by GPCR
Peptide ligand-binding receptors
cluster
path group 2
Neuronal System
Transmission across Chemical Synapses
Neurotransmitter receptors and postsynaptic signal transmission
Translation
mRNA Splicing - Major Pathway
mRNA Splicing
Processing of Capped Intron-Containing Pre-mRNA
Protein-protein interactions at synapses
Extracellular matrix organization
Eukaryotic Translation Initiation
Cilium Assembly
Organelle biogenesis and maintenance
Intraflagellar transport
Anchoring of the basal body to the plasma membrane
Mitotic Prometaphase
DNA strand elongation
Chromatin organization
Chromatin modifying enzymes
Polymerase switching
Cohesin Loading onto Chromatin
cluster
path group 3
Peptide chain elongation
Eukaryotic Translation Elongation
Viral mRNA Translation
Eukaryotic Translation Termination
Selenocysteine synthesis
Non-integrin membrane-ECM interactions
Extracellular matrix organization
ECM proteoglycans
Deubiquitination
Class I MHC mediated antigen processing & presentation
Laminin interactions
PTEN Regulation
Antigen processing: Ubiquitination & Proteasome degradation
Downstream TCR signaling
SLC-mediated transmembrane transport
DNA Repair
tRNA processing
tRNA processing in the nucleus
snRNP Assembly
Metabolism of non-coding RNA
cluster
path group 4
Neuronal System
Transmission across Chemical Synapses
Neurotransmitter receptors and postsynaptic signal transmission
Protein-protein interactions at synapses
Eukaryotic Translation Elongation
Peptide chain elongation
Eukaryotic Translation Termination
Viral mRNA Translation
Response of EIF2AK4 (GCN2) to amino acid deficiency
Neurexins and neuroligins
The citric acid (TCA) cycle and respiratory electron transport
Respiratory electron transport, ATP synthesis by chemiosmotic coupling, and heat production by uncoupling proteins.
Respiratory electron transport
Organelle biogenesis and maintenance
Mitochondrial translation termination
RAC1 GTPase cycle
Golgi Associated Vesicle Biogenesis
trans-Golgi Network Vesicle Budding
Sphingolipid metabolism
Neutrophil degranulation
cluster
count
neglog10_pval

b

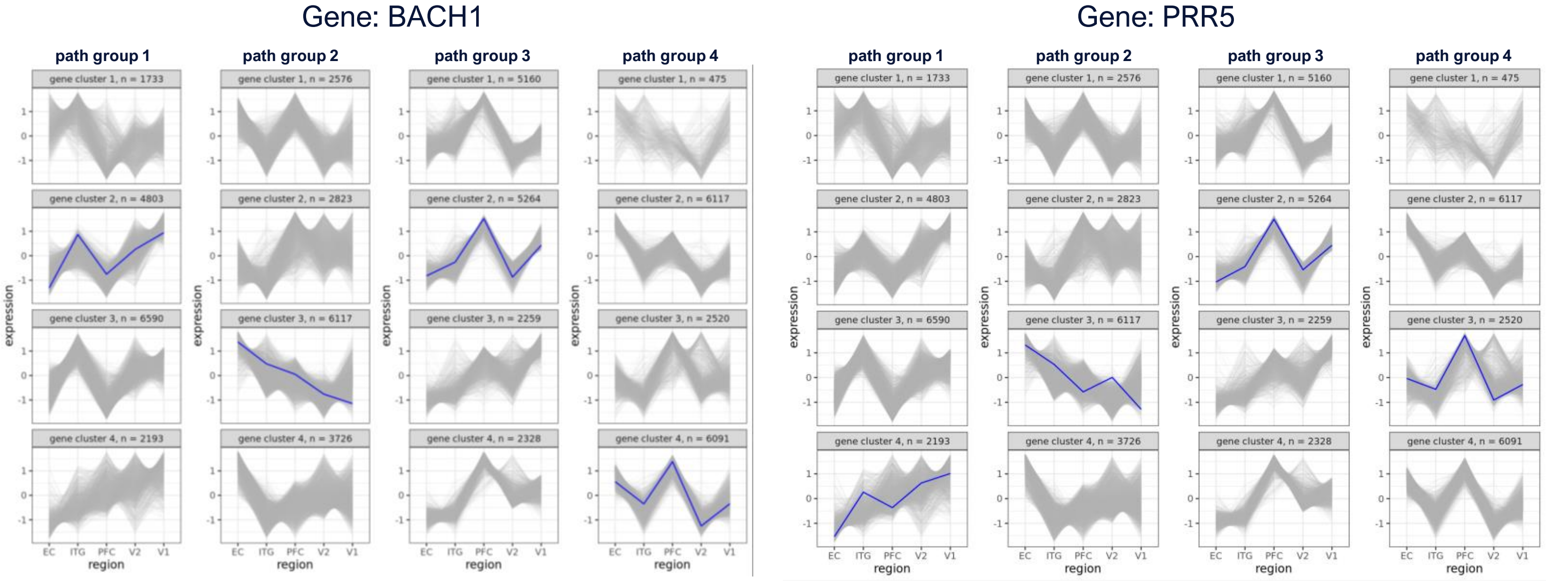

Gene: BACH1
Gene: PRR5
path group 1
path group 2
path group 3
path group 4
gene cluster 1, n = 1733
gene cluster 2, n = 4803
gene cluster 3, n = 6590
gene cluster 4, n = 2193
gene cluster 1, n = 2576
gene cluster 2, n = 2823
gene cluster 3, n = 6117
gene cluster 4, n = 3726
gene cluster 1, n = 5160
gene cluster 2, n = 5264
gene cluster 3, n = 2259
gene cluster 4, n = 2328
gene cluster 1, n = 475
gene cluster 2, n = 6117
gene cluster 3, n = 2520
gene cluster 4, n = 6091
expression
EC ITG PFC V2 V1
region
